# Antibody-Drug Conjugate Using Ionized Cys-Linker-MMAE as the Potent Payload Shows Optimal Therapeutic Safety

**DOI:** 10.3390/cancers12030744

**Published:** 2020-03-21

**Authors:** Yanming Wang, Lianqi Liu, Shiyong Fan, Dian Xiao, Fei Xie, Wei Li, Wu Zhong, Xinbo Zhou

**Affiliations:** National Engineering Research Center for the Emergency Drug, Beijing Institute of Pharmacology and Toxicology, Beijing 100850, China; yanming0117@163.com (Y.W.); llianqi@126.com (L.L.); fsyn1996@163.com (S.F.); be_xiaodian@163.com (D.X.); xiefei0058@163.com (F.X.); a_moon1096@163.com (W.L.)

**Keywords:** tumor, antibody-drug conjugate, linker, MMAE, Cys-linker-MMAE

## Abstract

Monomethyl auristatin E (MMAE) is the most popular and widely used cytotoxin in the development of antibody-drug conjugates (ADCs). However, current MMAE-based ADCs are all constructed using cleavable linkers, and this design concept still has insurmountable drawbacks. Their potential instabilities and lipophilic MMAE-induced “bystander effect” inevitably increase the toxicity to normal tissues. Herein, we overturn previous negative views of MMAE-based ADCs with non-cleavable linkers and propose using ionized *L*-Cysteine (Cys)-linker-MMAE as a novel payload, which can ingeniously enrich and enter tumor cells through receptor-mediated endocytosis of antibodies while its lower permeability helps to avoid further off-target toxicity. We demonstrate that Cys-linker-MMAE maintains high potency similar to free MMAE at the tubulin molecular level and can also be efficiently released in target cells. As a result, the preferred ADC (mil40-**15**) not only exhibits ideal plasma stability and maintains potent cytotoxicity as MMAE (IC_50_: 10^−11^ M), but also shows improved safety with lower bystander toxicity (IC_50_: 10^−9^ M), its maximum tolerated dose approaching the level of the naked antibody (160 mg/kg). This study indicated that Cys-linker-MMAE has the potential as a potent payload for ADCs, which is expected to provide novel strategies for the development of MMAE-based ADCs.

## 1. Introduction

Antibody-drug conjugates (ADCs), using antibodies to selectively deliver potent cytotoxins to tumor cells, have recently become a promising next-generation of anticancer drugs [1,2]. At present, seven ADCs (Mylotarg^®^, Adcetris^®^, Kadcyla^®^, Besponsa^®^, Polivy^®^, Padcev^®^, and Enhertu^®^) have been approved to treat many types of cancer, while at least 80 ADCs are in clinical trials [3,4,5].

Monomethyl auristatin E (MMAE), a synthetic analog of dolastatin 10 with high potency at the cellular level (IC_50_: 10^−11^–10^−9^ M) [6,7], is the most commonly used cytotoxins in nearly one-third of clinical ADCs [1,8]. The current MMAE-based ADCs operate mainly through the introduction of an enzyme-cleavable linker to ensure that free MMAE can act in its best active form, which can further improve their efficiency and response rate through “bystander killing effects” [9]. For example, the valine-citrulline (VC) dipeptide-based and glucuronides-based cleavable linker systems rely on 1,6-elimination of spacers to drive the release of free MMAE (Figure S1) [10]. At present, this self-immolative cleavable linker system has been successfully applied to the marketed ADCs such as Adcetris^®^, Polivy^®^, and Padcev^®^.

**Figure 1:**
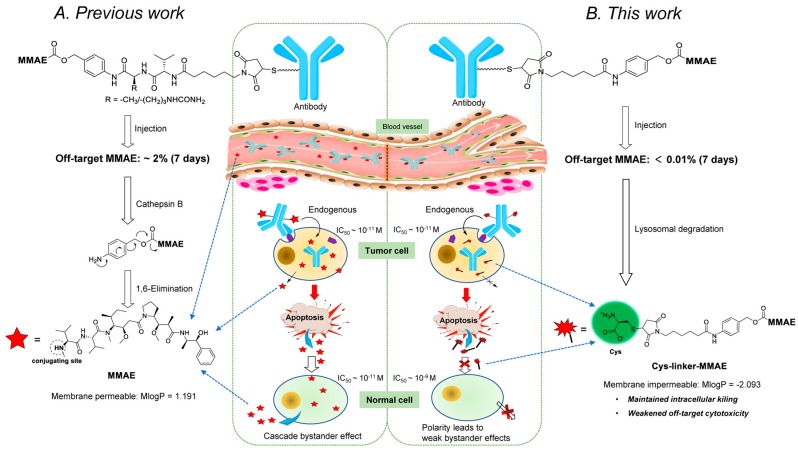
The drug release process of MMAE- and Cys-linker-MMAE-based ADCs. (**A**) The drug release mechanism of traditional MMAE-based ADCs with cathepsin B-cleavable dipeptide linkers. (**B**) The drug release mechanism of the novel Cys-linker-MMAE-based ADC and its cellular metabolites. Cys: *L*-cysteine; MMAE: monomethyl auristatin E; ADCs: antibody-drug conjugates; IC_50_: half maximal inhibitory concentration; M: mol/L; MlogP: Moriguchi octanol-water partition coefficient.

Although the current MMAE-based ADCs with cleavable linkers have achieved great success in clinical applications, there are still limitations in the design concept and the antitumor activity should always be balanced by safety considerations [5,11]. These ADCs work by releasing MMAE in its unmodified form under the catalysis of enzymes (cathepsin B, etc.) [12], which are ubiquitously expressed in most mammalian cells [13,14,15]. Moreover, it is well known that only <1% of the injected dose of ADCs targets tumors in patients [16,17,18]. In spite of the improved efficacy of the “bystander effects”, the permeable MMAE inevitably released in normal tissues and produced off-target toxicity (Figure 1A) [19]. Typically, most ADCs have obviously toxic effects before reaching their maximum effective dose [20]. In addition, researchers have demonstrated that dipeptide-based linker systems are unlikely to increase the therapeutic index due to their susceptibility to extracellular carboxylesterase [21]. How to further improve the therapeutic index of MMAE-based ADCs has become one of the main factors restricting their further development.

Herein, we propose the hypothesis of using non-cleavable *L*-Cysteine (Cys)-linker-MMAE as the potent payload directly. The ionized effector molecules skillfully use receptor-mediated endocytosis of the antibody to enter tumor cells and produce cumulative effects [11], meanwhile, their ionic characteristics effectively reduce toxicity by an accelerated in vivo clearance process and reduce penetration into normal cells (Figure 1B) [22]. Although this design strategy has been applied to other cytotoxins, such as DM1 which was used in the marketed Kadcyla^®^ [23], MMAE has rarely been modified in ADCs thus far, and the conclusions of the only relevant research were negative [24].

In this study, we provide favorable evidence for using Cys-linker-MMAE as a potent payload for the first time. Compared with traditional MMAE-based ADCs with cleavable linkers, the ADC with Cys-linker-MMAE as the payload returns to the simplest structure and has better plasma stability. The ADC (mil40-**15**) obtained by systematic optimization maintained cell inhibitory activity similar to that of MMAE (IC_50_ = 10^−11^ M), while its excellent stability and low “bystander effect” (IC_50_ = 10^−9^ M) provide significant safety advantages. Its maximum tolerable dose was close to the level of the naked antibody (160 mg/kg). This study is expected to provide a new strategy for the further development of MMAE-based ADCs, which is especially beneficial for the treatment of homogeneous tumors with high antigen expression.

## 2. Results

### 2.1. Design and Synthesis of the Cys-Linker-MMAE-Based ADC Payloads

We designed and synthesized a series of linker-MMAE constructs (**11**–**15**) based on different non-cleavable linkers. After these linker-MMAE constructs were modified on the Cys site of the antibodies, the resulting ADCs can be metabolized into stable Cys-linker-MMAE conjugates in the target cells to exert pharmacodynamic effects. Moreover, compound **16**, which can be cleaved by cathepsin B to release free MMAE, was prepared according to the method we reported previously [25] and was used to prepare a cleavable ADC as a control. The structures of the synthetic linker-MMAE conjugates are shown in Table 1.

To further characterize the “bystander effect” of these corresponding ionized Cys-linker-MMAE conjugates (Cys-**11**, Cys-**12**, Cys-**13**, Cys-**14**, and Cys-**15**), membrane permeability parameters were further calculated using ADMET Predictor 9.5 (Pharmogo Co., Shanghai, China), and the results obtained are shown in Table S1. The results showed that the Moriguchi octanol-water partition coefficients (MlogP values) of the conjugates were significantly reduced compared to MMAE, and the parameters characterizing permeability (S+Peff, S+MDCK, and Perm_Cornea) were also reduced, which indicates that the ionized Cys-linker-MMAE conjugate has lower membrane permeability. In addition, the difference between the parameters of Cys-**11**–Cys-**15** was not significant.

### 2.2. Tubulin Polymerization Inhibition Study

**Figure 2:**
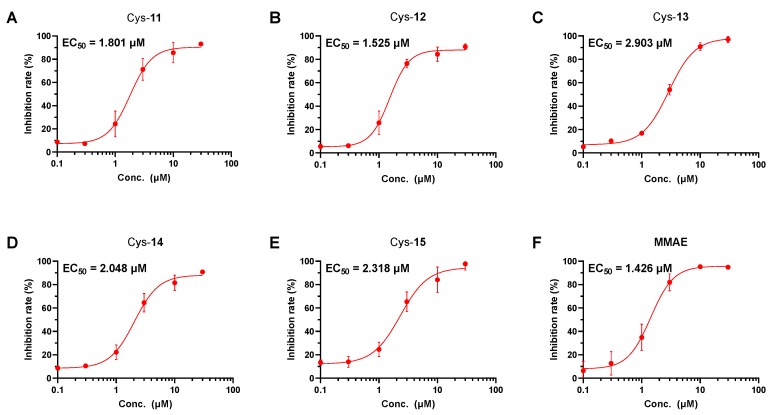
Tubulin polymerization inhibition analysis. (**A**) Cys-**11**, (**B**) Cys-**12**, (**C**) Cys-**13**, (**D**) Cys-**14**, (**E**) Cys-**15** and (**F**) MMAE were all tested at a range of concentrations (0.1, 0.3, 1.0, 3.0, 10.0, and 30.0 μM), and the inhibitory activity of tubulin polymerization is expressed as the concentration for 50% maximal effect (EC_50_). The results are shown as the mean ± standard deviation (SD) from triplicate experiments.

As a derivative of auristatin, MMAE exerts anti-tumor effects by inhibiting the polymerization of tubulin [26]. In this study, we used a molecular-level tubulin inhibition assay to rapidly assess the efficacy of ionized conjugates to circumvent the effects of the differences in membrane permeability. We evaluated the tubulin inhibitory activity of the synthesized Cys-linker-MMAE conjugates with free MMAE as a control. The results of the experiment showed that the effects of MMAE (EC_50_ = 1.426 μM) on tubulin were reduced when the *N*-terminus was modified with the linkers (Cys-**11**–Cys-**15**: EC_50_ = 1.525–2.903 μM), but this effect was relatively limited (Figure 2). All of the tested Cys-linker-MMAE conjugates produced concentration-dependent inhibition of tubulin polymerization, providing a basis for developing ADCs with Cys-linker-MMAE as potent payloads [27]. In addition, the results also show that the effect of linker length on the activity of the conjugate is weak, and the insertion of the *p*-aminobenzyl alcohol (PAB) structure in the linker does not significantly reduce its activity.

### 2.3. Evaluation of the Antibody-Drug Conjugates (ADCs) Preparation

Based on the consideration of reducing “bystander effects” and toxicity, we preferred to generate an ADC for further research by using conjugate **15** with relatively weak activity. We conjugated synthetic **15** to the anti-human epidermal growth factor receptor 2 (HER2) humanized antibody mil40 to produce a Cys-linker-MMAE based ADC. According to previous methods [25], the hydrophobic interaction chromatography (HIC)-HPLC spectrum demonstrated that the average drug-to-antibody ratio (DAR) of the conjugate product (mil40-**15**) was approximately 3.88, and the size exclusion chromatography (SEC)-HPLC spectrum demonstrated that the resulting monomeric product had a purity of >98%. In addition, the resulting ADC had an endotoxin content of less than 1 EU/mg, which was used for subsequent characterization and evaluation studies.

### 2.4. Stability Assays of the Conjugate in Plasma

**Figure 3:**
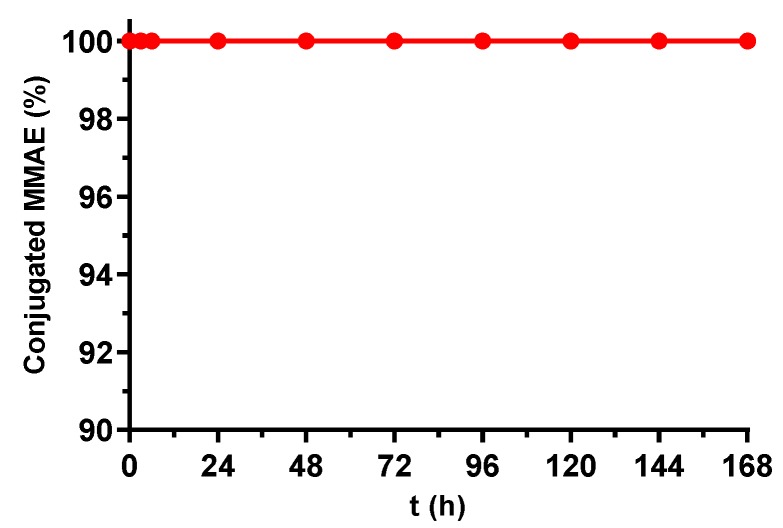
Stability of the Cys-linker-MMAE-based ADC in 50% plasma at 37 °C. The test concentration of mil40-**15** was 250 nM, and the lower limit of quantification (LLOQ) and upper limit of quantification (ULOQ) of MMAE were 2 nM and 250 nM, respectively. Each sample point contains two repetitions and the results are shown as the mean.

To assess the conjugate stability, mil40-**15** was incubated in two-fold diluted human plasma at 37 °C for 7 days. At set time points (0, 3, 6, 24, 48, 72, 96, 120, 144, and 168 h), samples were taken and the content of free MMAE was detected [28]. LC-MS/MS analysis of the samples showed that <0.01% of the MMAE (below the lower limit of quantification of 2 nM) of the ADC mil40-**15** (250 nM; the corresponding concentration of MMAE ~1000 nM) was released (Figure 3), indicating that mil40-**15** has very desirable plasma stability. ADCs based on Cys-linker-MMAE do not cause significant cytotoxicity, which would serve as an important basis for reducing toxicity and improving the safety of ADCs with MMAE as the payload.

### 2.5. Drug Release Study at the Cellular Level

To study the drug release characteristics of the obtained ADCs in cells, we first performed preliminary verification on the HER2 antigen-positive tumor cell lines BT-474 and NCI-N87. NCI-N87 and BT-474 cells (1.45 × 10^7^) were administered mil40-**15** at concentrations of 18.5 ng/mL and 52.5 ng/mL, respectively. After 24 h, LC-MS/MS analysis of the samples taken from NCI-N87 cells showed that the peak from MMAE was too low to be accurately detected, and the concentration of released MMAE in the samples taken from BT-474 was 0.0677 ng/mL (4.67 pg/mL/10^6^ cells), which means that only a very low proportion (<0.1%) of free MMAE was released at the cellular level.

Furthermore, NCI-N87 and BT-474 cells were suspended in 15 mL of medium containing 278 ng and 787 ng of mil40-**15**, respectively. After 24 h of incubation, the cell extracts were used for further analysis. As shown in Figure S2, two major metabolites (M1 and M2) of mil40-**15** were detected in both the BT474 and NCI-N87 samples. As a pair of optical isomers caused by the asymmetric synthesis of the maleimide group in linkers and the thiol in *L*-cysteine [29], the generate process of M1 and M2 is similar to that of Kadcyla^®^ producing two metabolites in *R* or *S* configuration at the carbon of the thioether bond [30]. Since the two metabolites of mil40-**15** have the same molecular mass as Cys-**15** (MS m/z [M+H]^+^ calculated for C_60_H_93_N_8_O_14_S 1181.65; found 1181.65), our results preliminarily indicate that mil40-**15** can successfully release Cys-linker-MMAE instead of free MMAE in target cells [24].

### 2.6. Flow Cytometry Analysis

In the previous tubulin inhibition study, we initially confirmed that Cys-**15** has relatively weaker activity than MMAE. Herein, we used flow cytometry to investigate whether the Cys-linker-MMAE based ADC mil40-**15** has the potential to induce tumor cell apoptosis and block the tumor cell cycle. Flow cytometry analysis initially indicated that the ratio of apoptotic and dead cells in the tested HER2-positive BT-474 and NCI-N87 cell lines increased in a concentration-dependent manner (Figure S3A). In addition, the results showed a significant increase in G_2_/M DNA content in a concentration-dependent manner (Figure S3B). These results suggest that mil40-**15** can induce cell apoptosis and produce potent G_2_/M arrest in BT-474 and NCI-N87 cells.

### 2.7. In Vitro Potency Assay of the ADC, Antibody, MMAE, and Cys-Linker-MMAE Conjugate

**Figure 4:**
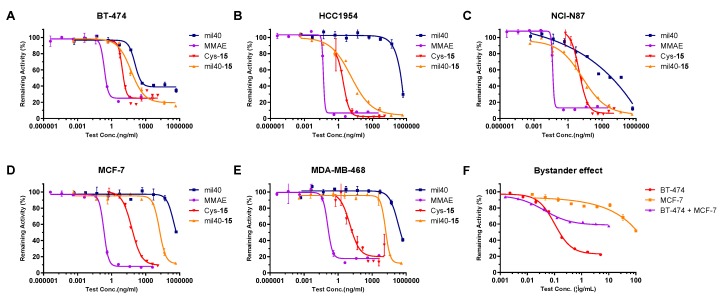
Cytotoxicity of the Cys-linker-MMAE-based ADC in tumor cells. (**A**) The cytotoxicity assays performed in BT-474 cell line. (**B**) The cytotoxicity assays performed in HCC1954 cell line. (**C**) The cytotoxicity assays performed in NCI-N87 cell line. (**D**) The cytotoxicity assays performed in MCF-7 cell line. (**E**) The cytotoxicity assays performed in MDA-MB-468 cell line. (**F**) The bystander effect test performed in BT-474 and MCF-7 cell lines. The results are shown as the mean ± SD from triplicate experiments.

To further evaluate the cytotoxic activity of mil40-**15**, HER2-positive cell lines (BT-474, HCC1954, and NCI-N87) and HER2-negative cell lines (MCF-7, and MDA-MB-468) were treated with ADC (mil40-**15**), with the naked antibody (mil40), cytotoxin (MMAE), and Cys-linker-MMAE (Cys-**15**) as controls [31]. As shown in Figure 4, mil40-**15** showed higher cytotoxicity and maximum inhibition rate compared with mil40 in all HER2-positive tumor cell lines, and the activity of mil40-**15** in antigen-negative cells can be significantly reduced by 279–1276-fold compared to the antigen-positive cells (Table 2). Even for HCC1954 cells that are not sensitive to mil40, ADCs had significant cytostatic activity. In comparison, MMAE had potent antitumor activity in both HER2-positive and HER2-negative cell lines with almost no antigenic selectivity (Table 2). In addition, the Cys-linker-MMAE conjugate (Cys-**15**) showed relatively weak activity in each cell line compared to MMAE (a decrease of approximately 22–177-fold), which may be mainly related to the decrease in cell membrane permeability of Cys-**15**.

Furthermore, we evaluated the “bystander effect” of mil40-**15** at the cellular level (Figure 4). We mixed antigen-positive BT-474 cells with antigen-negative MCF-7 cells in a 1:1 ratio, and the two individual cell lines of the same density were used for comparison [32,33]. The results showed that the efficacy of ADC on HER2^+^ and HER2^–^ cells in mixed culture is between the two single-cultured cells, and the maximum inhibition rates of mil40-**15** for the BT-474, MCF-7, and BT-474+MCF-7 cells were 78%, 48%, and 42%, respectively. Furthermore, the potency (IC_50_) of mil40-**15** in BT-474 cells was 92 and 914 times higher than that in BT-474 + MCF-7 and MCF-7 cells, respectively. The test shows that the ADC with Cys-linker-MMAE as payload has no significant “bystander effect”, which may be beneficial for improving the therapeutic index.

### 2.8. Trafficking Assay by Fluorescence Microscopy

**Figure 5:**
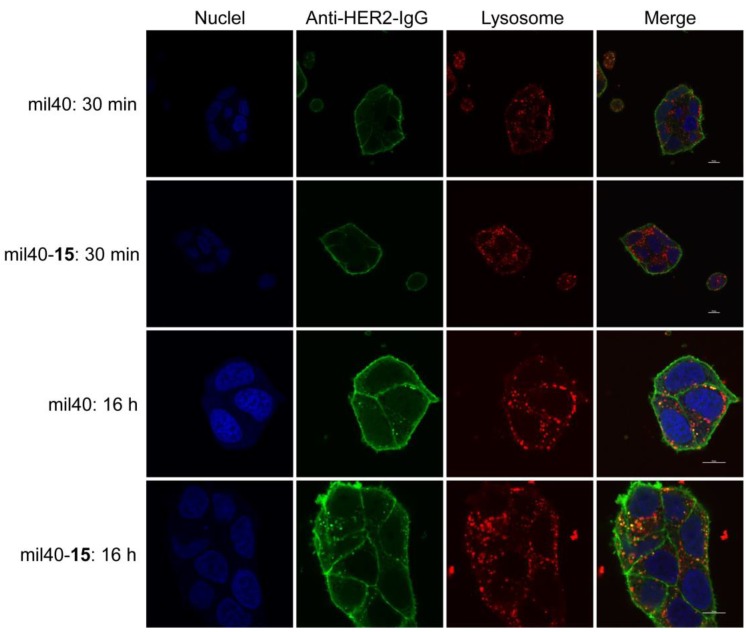
Receptor-mediated internalization of the Cys-linker-MMAE-based ADC in the HER2^+^ breast cancer cell line BT-474. Cells were treated at 4 °C with mil40 and mil40-**15** labeled with an Alexa Fluor 488-conjugated goat anti-human IgG antibody for 0.5 h and further incubated at 37 °C for 16 h. Scale bar = 10 μm.

In the BT-474 cell model, HER2-mediated endocytosis and intracellular trafficking of mil40-**15** were further elucidated by using laser scanning confocal microscopy. As shown in Figure 5, the cells were incubated at 4 °C for 30 min, and both the naked antibody and the ADC were bound to the cell peripheral membrane of BT-474 cells with the anti-HER2-IgG staining (green) that was not localized inside the cells or colocalized with lysosomal markers (red). Upon elevating the temperature to 37 °C for 16 h, mil40 and mil40-**15** internalized into the tumor cells and colocalized with the lysosomal markers (yellow), indicating that both the antibody and the ADC could be rapidly internalized into antigen-positive cells and continuously transported into the lysosomes for degradation.

### 2.9. In Vivo Potency in Xenografted Nude Mice

**Figure 6:**
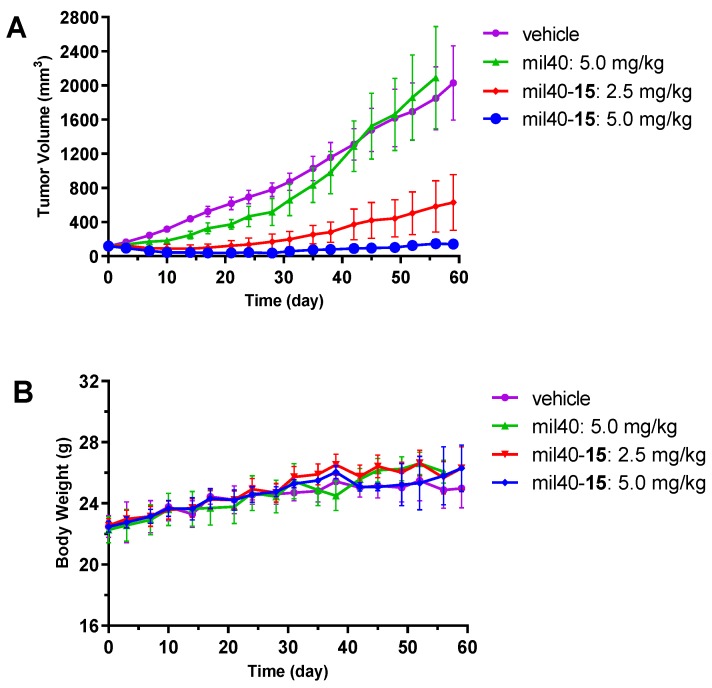
Xenograft studies of the Cys-linker-MMAE-based ADC. (**A**) The tumor volume of the test animals during the treatment and observation period. (**B**) Changes in the body weights of the mice during the observation period. Nude mice were implanted subcutaneously with NCI-N87 cells. When the tumors reached ~120 mm^3^, the animals were given vehicle, mil40, or ADC on days 0, 7, 14, and 21. The results are shown as the mean ± SD, *n* = 6/group.

An NCI-N87 xenograft model of HER2-positive gastric cancer cells in BALB/c nude mice was designed to assess the efficacy of mil40-**15** in vivo. The mice were given vehicle, mil40, or ADC on days 0, 7, 14, and 21. As shown in Figure 6A, the antitumor efficacy of ADC mil40-**15** showed significant dose-dependence, which had more significant tumor killing activity (*p* = 0.0179) and tumor growth inhibition (tumor inhibition rate = 93%) than the naked antibodies at the dose of 5 mg/kg. Moreover, the ADC treatment group showed persistent tumor growth inhibition during the one month observation period after discontinuation of the drug. In addition, during the treatment period and observation period after administration, no significant weight loss was seen in the mice of all treatment groups, indicating that mil40 and mil40-**15** were preliminarily well tolerated at the therapeutic dose (Figure 6B).

### 2.10. In Vivo Imaging of Fluorescein-Labeled ADC

**Figure 7:**
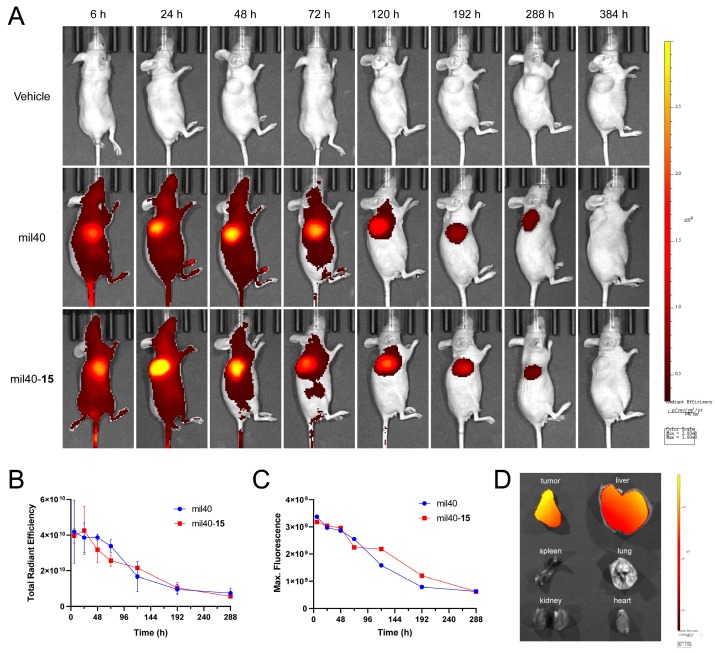
Fluorescence imaging of the Cys-linker-MMAE-based ADC in NCI-N87 xenograft model. (**A**) In vivo imaging of fluorescein-labeled ADC, color scale represents photons/s/cm^2^/steradian. (**B**) The total radiant efficiency of test mice in NCI-N87 xenograft model; the results are shown as the mean ± SD, *n* = 3/group. (**C**) The maximum fluorescence of test mice in NCI-N87 xenograft model. (**D**) In vitro fluorescence imaging of mouse tissues in ADC administration group.

The in vivo tissue distribution and targeting accumulation capability of mil40-**15** were evaluated in an NCI-N87 Balb/c-nude mice xenograft model via an optical molecular imaging system. After i.v. administration to the mice in the two Dylight 680-labeled groups at a dosage of 10 mg/kg, mil40 and mil40-**15** both showed higher fluorescence within 6 h and began to accumulate to the tumor. Then, an increase in the fluorescence signal of mil40 and mil40-**15** was initially visualized in the tumor sites within 24 h, and the image of tumor localization was clearly maintained for more than 12 days; however, the control group did not show any significant fluorescence (Figure 7A). As shown above, there was strong selective tumor accumulation and localization in the HER2-positive NCI-N87 tumors with naked antibody mil40 and mil40-**15**, indicating that ADC targets tumors as smoothly as naked antibody and further releases potent payload in tumor cells.

We further quantified the total fluorescence value and maximum fluorescence intensity of the tumors in the test animals. As shown in Figure 7B,C, there was no significant difference between mil40 and mil40-**15**, showing that ADC has pharmacokinetic properties similar to naked antibody. In addition, one mouse in the ADC dose group was randomly selected for tissue dissection and imaging in vitro after 32 h of administration. The results showed that the tumor tissue had the highest fluorescence intensity; however, except for the liver, the fluorescence values in the tissues such as the heart, spleen, lung, and kidney were all weak, indicating that ADC has good tissue distribution (Figure 7D).

### 2.11. Hematological Analysis

**Figure 8:**
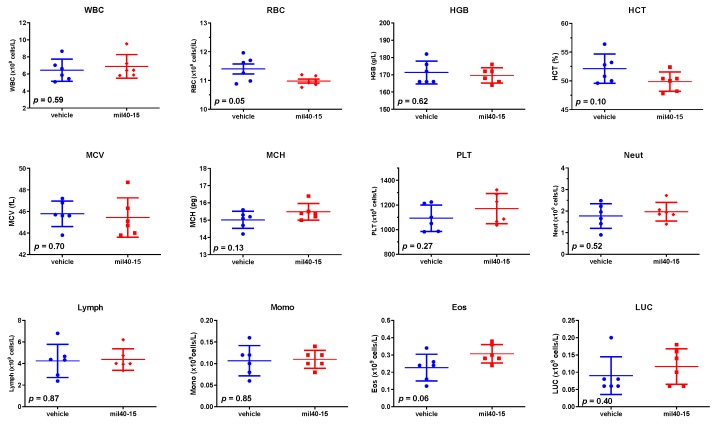
Hematological analysis of the Cys-linker-MMAE-based ADC. The results are shown as the mean ± SD, *n* = 6/group. WBCs: white blood cells; RBCs: red blood cells; HGB: hemoglobin; HCT: hematocrit; MCV: mean corpuscular volume; MCH: mean corpuscular hemoglobin; PLT: platelets; Neut: neutrophils; Lymph: lymphocytes; Momo: monocytes; Eos: eosinophils; LUC: large unstained cells.

In the xenograft model, this Cys-linker-MMAE-based ADC showed ideal tolerance at therapeutic doses. To further analyze the safety of the drug, hematological analysis was performed on mice in the high-dose ADC treatment group (5 mg/kg) with the vehicle group as a control. As shown in Figure 8, unpaired two-tailed *t*-tests of the hematological parameters of the blood samples showed that there was no significant difference between the ADC and vehicle treatment groups (*p* ≥ 0.05), indicating that the ADC does not produce significant hematologic toxicity at therapeutic doses.

### 2.12. In Vivo Tolerability Study

**Figure 9:**
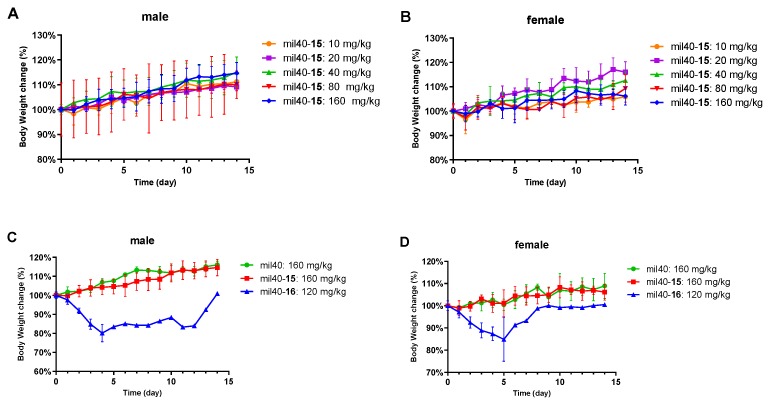
Tolerance study of the Cys-linker-MMAE-based ADC. (**A**) Body weight changes in male mice after administration of mil40-**15**. (**B**) Body weight changes in female mice after administration of mil40-**15**. (**C**) Body weight changes in male mice after administration of mil40, mil40-**15**, and mil40-**16**. (**D**) Body weight changes in female mice after administration of mil40, mil40-**15**, and mil40-**16**. The results are shown as the mean ± SD, *n* = 3/group.

We further studied the tolerance of mil40-**15** to verify the safety of this Cys-linker-MMAE-based ADC. The primary toxicity measurements were made in wild-type animals rather than tumor-bearing animals to distinguish between the effects due to treatment from those potentially caused by the tumors. Normal CD-1 mice were given a single treatment of mil40-**15** in a series of doses (10, 20, 40, 80, and 160 mg/kg) by intravenous injection. As shown in Figure 9, both male and female mice did not experience significant weight changes even at the highest dose. However, at a dose of 160 mg/kg, one animal in the ADC and antibody treatment groups developed mild alopecia on the right hindlimb or a scab on the dorsal neck, indicating that the two animals were administered near the maximum tolerated dose (MTD). As a comparison, mice in the dipeptide-based cleavable ADC (mil40-**16**) treatment group developed alopecia and scab symptoms at a dose of 80 mg/kg, and several mice began to exhibit decreased activity, haircoat piloerection, half-closed eyes, and death at 120 mg/kg. The above results show that the Cys-linker-MMAE-based ADC has a very wide therapeutic window, which is expected to further improve the therapeutic index of the MMAE-based ADC.

## 3. Discussion

Chemotherapy drugs still have a wide range of clinical applications, but their narrow therapeutic window limits their therapeutic benefits. ADCs are designed to use the targeting of antibodies to deliver small molecule drugs to tumor tissues while avoiding toxicity. This technology has enabled some highly active cytotoxins to be successfully used in clinical oncology treatments. However, the design of current MMAE-based ADCs still has the essential defect as mentioned in Section 1. This work proposes and further validates a novel ADC design strategy using Cys-linker-MMAE as the potent payload, aiming to overcome the ubiquitous toxicity and further increase its therapeutic window. We chose an anti-HER2 antibody mil40 (a biosimilar of Herceptin^®^) as a model carrier and the resulting ADC proved to maintain similar cell targeting and endocytosis characteristics.

First, we proved that our strategy meets the two basic requirements for the design of ADCs in terms of plasma stability and release payload in tumors. Our ADC is designed to be simpler in structure and the resulting product maintains high plasma stability with less than 0.01% MMAE shedding during a week. In addition, further colocalization and drug release studies indicate that mil40-**15** can effectively transfer into tumor cells by receptor-mediated endocytosis and release ionized Cys-**15** instead of permeable MMAE. As a comparison, it was reported by Neri et al. [34] that a non-internalized ADC with the same payload (Cys-**15**) hardly improved the in vivo efficacy even compared to the vehicle, meaning that the release of ionized payload inside target cells is necessary for effectiveness.

Further, we provide favorable evidence on the efficacy of ADCs with Cys-linker-MMAE as the payload. In the cytotoxicity test, although the IC_50_ value of ionized Cys-**15** can be reduced by two orders of magnitude compared to MMAE, we deduced that it is mainly caused by its poorer membrane permeability (MlogP values of Cys-**15** and MMAE are −2.093 and 1.191). Generally, the conventional cytotoxicity test cannot effectively reflect its true efficacy in ADC applications [11], which lead to some challenges in designing and screening the ionized cytotoxins of ADCs [35]. Herein, we applied a rapid screening method at the tubulin molecular level and confirmed that the efficacy of Cys-linker-MMAE conjugates did not significantly decrease compared to MMAE. Additionally, due to the poor permeability of Cys-**15** released intracellularly, it can be continuously enriched in target cells to enhance efficacy [11]; our cytotoxicity test showed that mil40-**15** maintains the same level of potency as free MMAE (IC_50_: 10^−11^ M). Eventually in the xenograft model, mil40-**15** showed significant antitumor activity at a dose of 2.5–5.0 mg/kg and can continuously inhibit tumor growth after discontinuation in the high-dose group.

Encouraged by the above data, we further conducted toxicity tests and confirmed that mil40-**15** has excellent safety advantages. Due to poor cell membrane permeability, Cys-**15** has lower cytotoxicity in vitro than MMAE, which means that Cys-**15** may have the potential to reduce off-target toxicity. It was validated in a subsequent “bystander effect” test that mil40-**15** efficiently killed target-positive BT-474 cells but was unable to kill an antigen-negative MCF-7 cell line, nor eradicate all cells in a mixture of the two cell types (killing effect reduced by about 100 times), indicating that Cys-**15** derived from apoptotic tumor cells will not have significant toxicity to surrounding normal cells. In addition, it was reported that ionized payloads can accelerate their clearance in the circulatory system [22], and Cys-**15** derived from off-target and apoptotic target cells may also reduce secondary damage. From the above points, mil40-**15** may be particularly beneficial for the treatment of antigen-homogeneous tumors, which is consistent with the non-cleavable ADCs that are based on other cytotoxins [19,36,37,38]. In the end, these advantages combined with excellent stability are reflected in the animal models. The preferred mil40-**15** demonstrated significant security features, including no significant weight loss and hematologic toxicity, while maintaining potent activity. The therapeutic window of mil40-**15** is twice as much as the traditional dipeptide-based ADC mil40-**16**, indicating that it may translate into a significant improvement in the therapeutic effect [39,40].

## 4. Materials and Methods

### 4.1. Reagents and Antibody

Unless otherwise indicated, all of the starting materials and solvents were obtained from commercial suppliers at the highest grade available and used without further purification. All reactions were monitored by thin layer chromatography (TLC) on silica gel plates (Yantai Dexin Bio-Technology Co., Ltd., Yantai, China) with fluorescence F-254 and visualized with UV light. Column chromatography was carried out on silica gel (200–300 mesh; Yantai Chemical Industry Research Institute, Yantai, China). Monomethyl auristatin E (MMAE) was purchased from Concortis Biosystems (San Diego, CA, USA). The anti-HER2 antibody mil40 (a biosimilar of Herceptin^®^) was purchased from Hisun Pharmaceutical Co., Ltd. (Taizhou, China).

### 4.2. Synthesis of the Linker-MMAE Conjugates

The synthetic route for the linker-MMAE conjugates is shown in Scheme 1. The original mass spectrometry and NMR spectra details of the synthesized compounds are provided in Figures S4–S28.

#### 4.2.1. Synthesis of 3-(2,5-Dioxo-2,5-Dihydro-1*H*-Pyrrol-1-yl)Propanoic Acid (**4**)

Compound **4** was synthesized using previously reported experimental protocols [25]. Maleic anhydride (16.51 g, 168.37 mmol) was added to a solution of compound **1** (10.0 g, 112.25 mmol) in AcOH (200 mL). The mixture was stirred at 120 °C for 6 h, and then the mixture was poured into water after cooling to room temperature. The solution was extracted with ethyl acetate (3 × 20 mL) and the combined organic layers were washed with brine, dried over anhydrous Na_2_SO_4_, and evaporated under reduced pressure to give the crude product. Purification was performed by silica column chromatography in 1:6 ethyl acetate/petroleum ether (v/v). A white solid was obtained in a yield of 86%. ^1^H-NMR (400 MHz, DMSO-*d6*): δ 12.37 (br, 1H), 7.03 (s, 2H), 3.61 (t, *J* = 7.31 Hz, 2H), 2.49 (t, *J* = 7.31 Hz, 2H). ESI m/z (M − H)^−^ calculated for C_7_H_7_NO_4_ 168.0; found 168.3.

#### 4.2.2. Synthesis of 6-(2,5-Dioxo-2,5-Dihydro-1*H*-Pyrrol-1-yl)Hexanoic Acid (**5**)

Compound **5** was synthesized with the experimental protocols described for compound **4** [25]. A white solid was obtained in a yield of 74%. ^1^H NMR (400 MHz, DMSO-*d6*): δ 11.98 (br, 1H), 7.01 (s, 2H), 3.39 (t, *J* = 7.30 Hz, 2H), 2.17 (t, *J* = 7.30 Hz, 2H), 1.51–1.44 (m, 2H), 1.24–1.17 (m, 2H). ESI m/z (M − H)^−^ calculated for C_7_H_7_NO_4_ 210.1; found 210.0.

#### 4.2.3. Synthesis of 3-(2-(2,5-Dioxo-2,5-Dihydro-1*H*-Pyrrol-1-yl)Ethoxy)Propanoic Acid (**6**)

Compound **6** was synthesized with the experimental protocols described for compound **4**. A white glassy solid was obtained with a crude product yield of 79%. The product was taken directly to the next reaction step without further purification. ESI m/z (M + H)^+^ calculated for C_9_H_12_NO_5_ 214.07; found 214.08; ESI m/z (M + Na)^+^ calculated for C_9_H_11_NNaO_5_ 236.05; found 236.05.

#### 4.2.4. Synthesis of 3-(2,5-Dioxo-2,5-Dihydro-1*H*-Pyrrol-1-yl)-*N*-(4-(Hydroxymethyl)Phenyl)Propanamide (**7**)

Compound **4** (1.0 g, 5.92 mmol) was dissolved in dry dichloromethane (DCM) (25 mL), and *p*-aminobenzyl alcohol (0.73 g, 5.92 mmol), 1-ethyl-(3-dimethylaminopropyl) carbodiimide hydrochloride (EDCI) (1.70 g, 8.88 mmol), and *N*,*N*′-diisopropylethylamine (DIPEA) (1.52 g, 11.84 mmol) were added. The reaction was stirred at room temperature for 12 h. After the completion of the reaction, the solvent was removed under reduced pressure to give a crude material, which was purified by column chromatography to obtain compound **7** as a white solid (0.53 g, 33% yield). ^1^H NMR (400 MHz, DMSO-*d6*): δ 9.96 (s, 1H), 7.47 (d, *J* = 8.68 Hz, 2H), 7.21 (d, *J* = 8.68 Hz, 2H), 7.03 (s, 2H), 5.10 (t, *J* = 5.76 Hz, 1H), 4.21 (d, *J* = 5.76 Hz, 2H), 3.70 (t, *J* = 7.14 Hz, 2H), 2.57 (t, *J* = 7.14 Hz, 2H). ^13^C-NMR (100 MHz, DMSO-*d6*): δ 170.83, 168.38, 137.63, 137.38, 134.65, 126.91, 119.01, 62.64, 35.04, 33.93. ESI m/z (M + H)^+^ calculated for C_14_H_15_N_2_O_4_ 275.10; found 275.10; ESI m/z (M + Na)^+^ calculated for C_14_H_14_N_2_NaO_4_ 297.09; found 297.09.

#### 4.2.5. Synthesis of 6-(2,5-Dioxo-2,5-Dihydro-1*H*-Pyrrol-1-yl)-*N*-(4-(Hydroxymethyl)Phenyl)Hexanamide (**8**)

Compound **8** was synthesized with the experimental protocols described for compound **7**. A light yellow solid powder was obtained in a yield of 55%. ^1^H NMR (400 MHz, DMSO-*d6*): δ 9.82 (s, 1H), 7.51 (t, *J* = 4.33 Hz, 2H), 7.21 (d, *J* = 8.70 Hz, 2H), 7.01 (s, 2H), 5.09 (t, *J* = 5.74 Hz, 1H), 4.41 (d, *J* = 5.60 Hz, 2H), 3.39 (t, *J* = 9.0 Hz, 2H), 2.26 (t, 2H), 1.59–1.47 (m, 4H), 1.24 (m, 2H). ESI m/z (M + H)^+^ calculated for C_17_H_21_N_2_O_4_ 317.15; found 317.15; ESI m/z (M + Na)^+^ calculated for C_17_H_20_N_2_NaO_4_ 339.13; found 339.13.

#### 4.2.6. Synthesis of 4-(3-(2,5-Dioxo-2,5-Dihydro-1*H*-Pyrrol-1-yl)Propanamido)Benzyl (4-Nitrophenyl) Carbonate (**9**)

Compound **7** (0.20 g, 0.73 mmol) was dissolved in dry *N*,*N*′-dimethylformamide (DMF) (25 mL), and bis(4-nitrophenyl) carbonate (0.27 g, 0.86 mmol) and DIPEA (0.13 g, 1.01 mmol) were added; the reaction was stirred at room temperature for 3 h. After the completion of the reaction, the solvent was concentrated under reduced pressure to remove the solvent to give a crude material, which was purified by column chromatography to obtain compound **9** as a light yellow solid (0.20 g, 63% yield). ^1^H NMR (400 MHz, DMSO-*d6*): δ 10.19 (s, 1H), 8.32 (d, *J* = 9.24 Hz, 2H), 7.60–7.56 (m, 4H), 7.39 (d, *J* = 8.40 Hz, 2H), 7.03 (s, 2H), 5.24 (s, 2H), 3.72 (t, *J* = 7.01 Hz, 2H), 2.60 (t, *J* = 7.01 Hz, 2H). ^13^C-NMR (100 MHz, DMSO-*d6*): δ 170.82, 168.71, 155.31, 151.99, 145.17, 139.49, 134.63, 129.46, 129.22, 125.42, 122.66, 119.14, 70.28, 35.10, 33.87. ESI m/z (M + H)^+^ calculated for C_21_H_18_N_3_O_8_ 440.11; found 440.12; ESI m/z (M + Na)^+^ calculated for C_21_H_17_N_3_NaO_8_ 462.09; found 462.11.

#### 4.2.7. Synthesis of 4-(6-(2,5-Dioxo-2,5-Dihydro-1*H*-Pyrrol-1-yl)Hexanamido)Benzyl (4-Nitrophenyl) Carbonate (**10**)

Compound **10** was synthesized with the experimental protocols described for compound **9**. A white solid was obtained in a yield of 65%. ^1^H-NMR (400 MHz, DMSO-*d6*): δ 9.98 (s, 1H), 8.32 (dt, *J* = 9.22 Hz, 2H), 7.62 (d, *J* = 8.70 Hz, 2H), 7.57 (dt, *J* = 9.22 Hz, 2H), 7.39 (d, *J* = 8.41 Hz, 2H), 7.01 (s, 2H), 5.23 (s, 2H), 3.40 (t, *J* = 7.0 Hz, 2H), 2.29 (t, *J* = 7.32 Hz, 2H), 1.58 (m, 2H), 1.51 (m, 2H), 1.25 (m, 2H). ESI m/z (M + H)^+^ calculated for C_24_H_23_N_3_O_8_ 482.16; found 482.16; ESI m/z (M + Na)^+^ calculated for C_24_H_23_N_3_NaO_8_ 504.14; found 504.14.

#### 4.2.8. Synthesis of (*S*)-2-((*S*)-2-(3-(2,5-Dioxo-2,5-Dihydro-1*H*-Pyrrol-1-yl)-*N*-Methylpropanamido)-3-Methylbutanamido)-*N*-((3*R*,4*S*,5*S*)-1-((*S*)-2-((1*R*,2*R*)-3-(((1*S*,2*R*)-1-Hydroxy-1-Phenylpropan-2-yl)amino)-1-Methoxy-2-Methyl-3-Oxopropyl)Pyrrolidin-1-yl)-3-Methoxy-5-Methyl-1-Oxoheptan-4-yl)-*N*,3-Dimethylbutanamide (**11**)

Compound **4** (35.5 mg, 0.21 mmol) was dissolved in dry DCM (25 mL), and MMAE (100.0 mg, 0.14 mmol), diethyl cyanophosphonate (46.0 mg, 0.28 mmol), and DIPEA (54.0 mg, 0.42 mmol) were added; the reaction was stirred at room temperature for 2 h. After the completion of the reaction, the solvent was removed under reduced pressure to give a crude material, which was purified by column chromatography to obtain compound **11** as a light yellow solid (72.0 mg, 56% yield). ^1^H NMR (400 MHz, DMSO-*d6*): δ 8.49 (t, *J* = 8.96 Hz, 0.5H), 7.89 (t, *J* = 8.40 Hz, 1H), 7.64 (d, *J* = 8.12 Hz, 0.5H), 7.31–7.18 (m, 5H), 7.01 (s, 2H), 5.38 (dd, *J* = 5.08 Hz, 1H), 4.75–4.35 (m, 3H), 4.04–3.77 (m, 3H), 3.62 (m, 3H), 3.39 (m, 3H), 3.30 (m, 1H), 3.25–3.12 (m, 8H), 3.07–2.79 (m, 5H), 2.73–2.57 (m, 2H), 2.42 (m, 1H), 2.29–1.92 (m, 4H), 1.81–1.72 (m, 3H), 1.54–1.48 (m, 2H), 1.30–1.24 (m, 1H), 1.11–0.93 (m, 6H), 0.89–0.71 (m, 17H). High resolution mass spectrometry (HR-MS) (ESI) m/z (M + H)^+^ calculated for C_46_H_73_N_6_O_10_ 869.5388; found 869.5383; HR-MS (ESI) m/z (M + Na)^+^ calculated for C_46_H_72_N_6_NaO_10_ 891.5208; found 891.5199.

#### 4.2.9. Synthesis of 6-(2,5-Dioxo-2,5-Dihydro-1*H*-Pyrrol-1-yl)-*N*-((*S*)-1-(((*S*)-1-(((3*R*,4*S*,5*S*)-1-((*S*)-2-((1*R*,2*R*)-3-(((1*S*,2*R*)-1-Hydroxy-1-Phenylpropan-2-yl)Amino)-1-Methoxy-2-Methyl-3-Oxopropyl)Pyrrolidin-1-yl)-3-Methoxy-5-Methyl-1-Oxoheptan-4-yl)(Methyl)Amino)-3-Methyl-1-Oxobutan-2-yl)Amino)-3-Methyl-1-Oxobutan-2-yl)-*N*-Methylhexanamide (**12**)

Compound **12** was synthesized with the experimental protocols described for compound **11**. A white solid was obtained in a yield of 63%. ^1^H NMR (400 MHz, DMSO-*d6*): δ 8.58 (m, 0.5H), 7.90 (m, 0.5H), 7.78–7.63 (m, 1H), 7.32–7.24 (m, 4H), 7.17 (m, 1H), 7.01 (s, 2 H), 5.38 (dd, *J* = 5.04 Hz, 1H), 4.64 (m, 1H), 4.54–4.37 (m, 2H), 3.99 (m, 2H), 3.78 (d, *J* = 10.36 Hz, 0.5H), 3.55 (m, 1H), 3.49 (m, 0.5H), 3.40–3.34 (m, 2H), 3.30 (m, 4H), 3.25–3.17 (m, 7H), 3.13–2.82 (m, 6H), 2.44–2.23 (m, 4H), 2.19–2.08 (m, 2H), 2.02–1.97 (m, 1H), 1.80 (m, 2H), 1.50 (m, 6H), 1.34–1.21 (m, 4H), 1.06–0.92 (m, 6H), 0.88–0.70 (m, 17H). HR-MS (ESI) m/z (M + H)^+^ calculated for C_49_H_79_N_6_O_10_ 911.5858; found 911.5858; HR-MS (ESI) m/z (M + Na)^+^ calculated for C_49_H_78_N_6_NaO_10_ 933.5677; found 933.5669.

#### 4.2.10. Synthesis of 4-(3-(2-(2,5-Dioxo-2,5-Dihydro-1*H*-Pyrrol-1-yl)Ethoxy)Propanamido)Benzyl ((S)-1-(((*S*)-1-(((3*R*,*4S*,5*S*)-1-((*S*)-2-((1*R*,2*R*)-3-(((1*S*,2*R*)-1-Hydroxy-1-Phenylpropan-2-yl)Amino)-1-Methoxy-2-Methyl-3-Oxopropyl)Pyrrolidin-1-yl)-3-Methoxy-5-Methyl-1-Oxoheptan-4-yl)(Methyl)Amino)-3-Methyl-1-Oxobutan-2-yl)Amino)-3-Methyl-1-Oxobutan-2-yl)(Methyl)Carbamate (**13**)

Compound **13** was synthesized with the experimental protocols described for compound **11**. A white solid was obtained in a yield of 55%. ^1^H NMR (400 MHz, DMSO-*d6*): δ 8.50 (m, 0.5H), 7.92 (d, 1H), 7.64 (d, 0.5H), 7.25–7.32 (m, 5H), 7.15–7.19 (m, 1H), 7.03 (s, 1H), 3.56–3.65 (m, 3H), 3.34 (m, 3H), 3.17–3.24 (m, 10H), 3.12 (m, 2H), 2.97 (m, 1H), 2.82–2.91 (m, 3H), 1.71–1.80 (m, 2H), 1.22–1.28 (m, 3H), 0.70–1.05 (m, 40H). HR-MS (ESI) m/z (M + H)^+^ calculated for C_48_H_77_N_6_O_11_ 913.5650; found 913.5645; HR-MS (ESI) m/z (M + Na)^+^ calculated for C_48_H_76_N_6_NaO_11_ 935.5470; found 935.5461.

#### 4.2.11. Synthesis of 4-(3-(2,5-Dioxo-2,5-Dihydro-1*H*-Pyrrol-1-yl)Propanamido)Benzyl ((*S*)-1-(((*S*)-1-(((3*R*,4*S*,5*S*)-1-((*S*)-2-((1*R*,2*R*)-3-(((1*S*,2*R*)-1-Hydroxy-1-Phenylpropan-2-yl)Amino)-1-Methoxy-2-Methyl-3-Oxopropyl)Pyrrolidin-1-yl)-3-Methoxy-5-Methyl-1-Oxoheptan-4-yl)(Methyl)Amino)-3-Methyl-1-Oxobutan-2-yl)Amino)-3-Methyl-1-Oxobutan-2-yl)(Methyl)Carbamate (**14**)

Compound **9** (67.0 mg, 0.15 mmol) was dissolved in dry DMF (5 mL), and MMAE (100.0 mg, 0.14 mmol), 1-hydroxybenzotriazole (HOBt) (25.0 mg, 0.19 mmol), and DIPEA (48.0 mg, 0.37 mmol) were added; the reaction was stirred at room temperature overnight. After the completion of the reaction, the solvent was removed under reduced pressure to give a crude material, which was purified by column chromatography to obtain compound **14** as a light yellow solid (70.0 mg, 50% yield). ^1^H NMR (400 MHz, DMSO-*d6*): δ 10.03 (s, 1H), 8.32 (d, *J* = 8.34 Hz, 0.5H), 8.06 (d, *J* = 8.34 Hz, 0.5H), 7.90 (d, *J* = 8.72 Hz, 0.5H), 7.64 (d, *J* = 8.72 Hz, 0.5H), 7.51 (dd, *J* = 2.24 Hz, 2H), 7.32–7.25 (m, 6H), 7.18 (m, 1H), 7.02 (s, 2H), 5.37 (dd, *J* = 5.04 Hz, 1H), 5.10–4.96 (m, 2H), 4.74–4.63 (m, 1H), 4.52–4.37 (m, 2H), 4.27 (t, *J* = 11.50 Hz, 1H), 4.00 (m, 2H), 3.71 (t, *J* = 7.10 Hz, 2H), 3.58–3.47 (m, 1H), 3.23–3.12 (m, 9H), 2.98–2.83 (m, 4H), 2.57 (t, *J* = 7.10 Hz, 2H), 2.41–2.39 (m, 1H), 2.29–2.23 (m, 1H), 2.14–1.92 (m, 3H), 1.77 (m, 3H), 1.52 (m, 2H), 1.33–1.23 (m, 2H), 1.05–0.97 (m, 7H), 0.89–0.75 (m, 19H). HR-MS (ESI) m/z (M + H)^+^ calculated for C_54_H_80_N_7_O_12_ 1018.5865; found 1018.5856; HR-MS (ESI) m/z (M + Na)^+^ calculated for C_54_H_79_N_7_NaO_12_ 1040.5684; found 1040.5678.

#### 4.2.12. Synthesis of 4-(6-(2,5-Dioxo-2,5-Dihydro-1*H*-Pyrrol-1-yl)Hexanamido)Benzyl ((*S*)-1-(((*S*)-1-(((3*R*,4*S*,5*S*)-1-((*S*)-2-((1*R*,2*R*)-3-(((1*S*,2*R*)-1-Hydroxy-1-Phenylpropan-2-yl)Amino)-1-Methoxy-2-Methyl-3-Oxopropyl)Pyrrolidin-1-yl)-3-Methoxy-5-Methyl-1-Oxoheptan-4-yl)(Methyl)Amino)-3-Methyl-1-Oxobutan-2-yl)Amino)-3-Methyl-1-Oxobutan-2-yl)(Methyl)Carbamate (**15**)

Compound **15** was synthesized with the experimental protocols described for compound **14**. A white solid was obtained in a yield of 90%. ^1^H NMR (400 MHz, DMSO-*d6*): δ 9.87 (s, 1H), 8.30 (m, 0.5H), 8.06 (m, 0.5H), 7.90 (d, *J* = 8.72 Hz, 0.5H), 7.64 (d, *J* = 8.72 Hz, 0.5H), 7.55 (d, *J* = 8.12 Hz, 2H), 7.32–7.24 (m, 6H), 7.18 (m, 1H), 7.00 (s, 2H), 5.38 (dd, *J* = 5.07 Hz, 1H), 5.09–4.96 (m, 2H), 4.69 (m, 1H), 4.50–4.39 (m, 2H), 4.26 (t, *J* = 11.34 Hz, 1H), 3.98 (m, 2H), 3.78 (d, *J* = 9.52 Hz, 0.5H), 3.55 (m, 1H), 3.48 (m, 0.5H), 3.39 (t, *J* = 7.14 Hz, 2H), 3.30 (m, 2H), 3.24–3.12 (m, 8H), 3.07–2.81 (m, 4H), 2.47 (m, 2H), 2.43–2.39 (m, 1H), 2.29-2.23 (m, 3H), 2.14–1.92 (m, 2H), 1.76 (m, 3H), 1.61–1.47 (m, 5H), 1.36–1.20 (m, 5H), 1.05–0.97 (m, 6H), 0.89–0.75 (m, 17H). HR-MS (ESI) m/z (M + H)^+^ calculated for C_57_H_86_N_7_O_12_ 1060.6334; found 1060.6324; HR-MS (ESI) m/z (M + Na)^+^ calculated for C_17_H_21_N_2_O_4_ 1082.6154; found 1082.6145.

### 4.3. Microtubule Polymerization Assay

We used the Tubulin Polymerization Assay Kit (Cat. # BK011P, Cytoskeleton, Inc., Denver, CO, USA) according to the manufacturer’s instructions for this fluorescence-based test. Before this test, the linker-MMAE conjugates were added to an *L*-cysteine solution (0.3 mmol/L in H_2_O, 900 μL), and the mixture was incubated for 10 min at room temperature for the conversion to Cys-linker-MMAE conjugates according to previously reported methods [8,14]. The potential metabolites (Cys-**11**, Cys-**12**, Cys-**13**, Cys-**14**, and Cys-**15**) of the ADCs were evaluated for their inhibitory activity on microtubule polymerization at different concentrations (0.1, 0.3, 1, 3, 10, and 30 μM) with MMAE and paclitaxel as controls.

### 4.4. Preparation of Reagents and Antibody-Drug Conjugates

The ADC was prepared according to previously reported methods [25]. To a solution of the antibody mil40 (5 mg/mL) in *L*-histidine buffer (20 mM, pH ≈ 7.5), a solution of tris(2-carboxyethyl)phosphine hydrochloride (TCEP; 2.3 equivalents) was added and the reaction was carried out for 90 min. Then, to this reduced antibody solution was added the maleimide derivative (**15**, 2 equivalents/SH group) in dimethylacetamide (DMAC) (6% v/v). After 120 min, the reaction was quenched with excess *N*-acetyl-*L*-cysteine (NAC; 8.0 equivalents) for 30 min. The mixture was adjusted to be weakly acidic (pH ≈ 5.5) with dilute acetic acid (0.3 mol/L) and placed on ice before buffer exchange by elution through Sephadex G25. Finally, the concentration of the target ADC (mil40-**15**) was determined by UV absorption at 280 nm, filtered through a 0.2 μm filter under sterile conditions, and stored at −80 °C before use for analysis and testing.

### 4.5. Characterization of the ADC

The aggregation of the final ADC was evaluated by size exclusion chromatography (SEC), and the drug-to-antibody ratio (DAR) was measured by hydrophobic interaction chromatography (HIC). SEC analyses were carried out on a 1260 HPLC (Agilent Technologies, Santa Clara, CA, USA) and TSK-gel G3000SWXL analytical column (7.8 mm × 300 cm; TOSOH Bioscience, Tokyo, Japan). The column was equilibrated with 0.22 μm-filtered 40 mM sodium phosphate and 150 mM sodium chloride at pH 7 at a flow rate of 0.5 mL/min, and the UV absorbance at 280 nm was detected for 35 min. HIC analyses were carried out on a TSK-gel Butyl-NPR column (2.5 μm, 4.6 mm × 3.5 cm, TOSOH Bioscience) in HIC buffer A (50 mM potassium phosphate, pH 7.0, and 1.5 M ammonium sulfate) and HIC buffer B (50 mM potassium phosphate, pH 7.0, 20% isopropanol). The gradient was 0% to 100% buffer B over 15 min, flow rate was 0.8 mL/min, and the UV detection wavelength was 280 nm. In addition, the endotoxin levels were determined using tachypleus amebocyte lysate (0.125 EU/mL, Zhanjiang bokang marine biological CO., LTD, Zhanjiang, China) according to the manufacturer’s instructions.

### 4.6. Stability Assays of the ADC in Plasma

Human plasma was obtained from Bioreclamation IVT (lot# BRH1343165, pH = 7.72, Westbury, NY, USA). The ADC solution (0.012 mM, 50 µL) was spiked into 2-fold diluted plasma (2350 µL) to reach a final concentration of 250 nM, and 50 µL aliquots of the spiked 2-fold diluted plasma were added to new tubes for different time points including 3, 6, 24, 48, 72, 96, 120, 144, and 168 h and incubated in an incubator at 37 °C. The assay was performed in duplicate, and the reaction was stopped by the addition of 300 µL of room temperature quench solution (methanol containing internal standards (IS; 100 nM alprazolam, 500 nM labetalol, and 2 µM ketoprofen) to the spiked plasma samples at the appointed time points. The samples were then vortexed for 5 min, and the samples in a plate were centrifuged at 3220× *g* for 30 min at 4 °C to precipitate the protein. Then, 50 mL of the supernatant were transferred to a new plate. The supernatant was diluted with water (150 μL), mixed well, and analyzed using LC-MS/MS (Agilent Technologies, Santa Clara, CA, USA).

### 4.7. Cellular Drug Release Process

The human HER2-positive cell lines, NCI-N87 and BT-474, which were purchased from the American Type Culture Collection (ATCC, Manassas, VA, USA), divided into vehicle and ADC dosing groups, and each group included two replicates. NCI-N87 and BT-474 cells were treated with medium containing 278 ng and 787 ng of the test drug, respectively. After 24 h of incubation, the medium was discarded, and the cells were separated by trypsin/ethylenediaminetetraacetic acid (EDTA), and the trypsin was inactivated by the addition of medium containing excess serum. Then, 10 mL of medium were added, followed by centrifugation at 120× *g* for 5 min, the cell supernatant was discarded, and 6 mL of cold methanol were added to the cell pellet extracts. The suspensions were kept at −20 °C for 30 min and centrifuged at 13,000× *g* for 20 min. Supernatants were evaporated by nitrogen gas blowing, and the resulting residues were redissolved in 600 μL of methanol containing an internal standard (IS = 100 nM alprazolam). To assess the amount of MMAE released from the ADC in NCI-N87 and BT474 cells, the samples were analyzed by LC-MS/MS.

### 4.8. Evaluation of the ADC for Tumor Cell Killing In Vitro

Human HER2-positive cell lines (BT-474, HCC1954, and NCI-N87) and HER2-negative cell lines (MCF-7 and MDA-MB-468) were purchased from the ATCC. Tumor cells were cultured in Roswell Park Memorial Institute (RPMI)-1640 medium (Cellgro, Manassas, VA, USA) supplemented with 10% fetal bovine serum (Invitrogen, Carlsbad, CA, USA) and GlutaMAX (Invitrogen, Carlsbad, CA, USA). Cells (3.3 × 10^4^ cells/well) were added to each well of 384-well plates and incubated at 37 °C overnight, after which 10 μL of compound aliquots were added to the assay plate. The plate was incubated for 7 days at 37 °C, 5% CO_2_, and 95% humidity. Then, the plates were removed from the incubator and equilibrated at room temperature for 10 min. The Cell Titer Glo^®^ (CTG, Promega, Madison, WI, USA) reagents were incubated at 37 °C before the experiment. The buffer was equilibrated to room temperature and used to dissolve the substrate. Then, 40 μL of CTG reagent was added to each well for detection (at 1:1 ratio to the culture medium). Luminescence was detected using an EnSpire plate reader, and Prism5 for Windows (GraphPad software, Inc., La Jolla, CA, USA) was used for data analysis, including the 50% inhibition concentration (IC_50_) calculations.

### 4.9. Confocal Analysis for Intracellular Localization

The Zenon™ pHrodo™ iFL red human IgG labeling reagent (Z25612), NucBlue™ Live ReadyProbes™ reagent (R37605), and CellLight™ Lysosomes-GFP, BacMam 2.0 (C10507) were purchased from Invitrogen (Carlsbad, CA, USA). BT-474 cells were seeded at a density of 2 × 104 cells/well in 24-well plates containing glass covers and lips and incubated at 37 °C overnight. Then, the cells were treated with 5 μg/mL of mil40 or mil40-**15** for 16 h at 37 °C. After washing the wells with ice-cold phosphate buffered solution (PBS), the cells were exposed on the chamber coverslip by a Cytospin, fixed with 4% paraformaldehyde for 10 min, and permeabilized with 0.2% Triton X-100 for 5 min. The mil40 and mil40-**15** present were detected with an Alexa Fluor 488-labeled goat anti-human IgG antibody. The lysosomes were labeled with a lysosomal-associated membrane protein 1 (LAMP-1) antibody followed by an Alexa Fluor 568-labeled goat anti-mouse IgG (H + L). The cell nuclei were stained with 4′,6-diamidino-2-phenylindole (DAPI). Fluorescence images were obtained using a Nikon A1 confocal microscope (Nikon, Corp., Tokyo, Japan).

### 4.10. Cell Cycle Arrest and Apoptosis Analysis

To evaluate the cell apoptosis in various types of cells, HER2-positive cell lines (BT-474, and NCI-N87) were seeded at a density of 2 × 10^6^ cells/well in 12-well plates, which were exposed to mil40-**15** at various concentrations (1, 10, and 100 μg/mL) for 24 h. Apoptosis and cell death were detected using an Annexin V-FITC Apoptosis Kit (Dojindo, Japan) and propidium iodide (PI) staining with a FACSCalibur (BD Biosciences, Franklin Lake, NJ, USA). For the cell cycle position analysis after drug exposure, the cells were treated as described above and allowed to incorporate bromodeoxyuridine (BrdUrd; Beyotime Biotechnology, Shanghai, China) for 20 min. Nascent DNA synthesis was detected with anti-BrdUrd fluoresceine isothiocyanate (FITC), and total DNA content was detected with PI. The cell cycle position and apoptosis analyses were measured with a FACSCalibur (BD Biosciences).

### 4.11. In Vivo Antitumor Activity in Human Gastric Xenograft Tumors

Female BALB/c nude mice (6–8 weeks old; approximately 22 g) were purchased from Beijing Ankaiyibo Biological Technology Co. Ltd. (Beijing, China), and were acclimated for 1 week before the experiment. The mice were inoculated subcutaneously with approximately 5 × 10^6^ NCI-N87 gastric cancer cells in 0.2 mL of serum-free RPMI-1640 medium for tumor development. When the tumors reached a volume of ~120 mm^3^, the mice were randomly divided into treatment and vehicle control groups (*n* = 6/group). The mice were given ADC (mil40-**15**), antibody (mil40), and vehicle (physiological saline) on days 0, 7, 14, and 21. The tumor volumes were monitored 2–3 times per week during the treatment period, and were calculated using the formula: TV = a × b^2^/2, where “a” and “b” are the long and short diameters of each tumor, respectively. All animal studies were performed according to the guidelines approved by the Institutional Animal Care and Use Committee (IACUC) of Pharmaron Co., Ltd. following the guidance of the Association for Assessment and Accreditation of Laboratory Animal Care (AAALAC) under license number ON-CELL-XEM-06012017 on 1 June 2017.

### 4.12. In Vivo Fluorescence Imaging Experiment

The in vivo fluorescence imaging experiments were investigated using an NCI-N87 Balb/c-nude mice xenograft model. The naked antibody mil40 and the ADC mil40-**15** were labeled with DyLight 680 according to the manufacturer’s instructions (Dylight 680 Antibody Labeling Kit, Thermo Fisher Scientific). When the tumor size reached approximately ~400 mm^3^, mice in the two groups were injected via the tail veins with the dose of 10 mg/kg, while an equal volume of vehicle was injected as a control. The mice were imaged under anesthesia at the indicated time points (6, 24, 48, 72, 120, 192, 288, and 360 h) using the IVIS^®^ SpectrumTM imaging system (PE, Waltham, MA, USA), and the data were analyzed using Living Image^®^ software (version number: 4.3.1, Waltham, MA, USA).

### 4.13. Hematological Analysis during Treatment

One week after the end of administration of the compounds to the NCI-N87 xenograft model (day 28), the animals were subjected to blood collection (100–180 μL) for whole blood analysis. For smaller blood samples, the volume was diluted 2-fold, and the whole blood count was performed, with the number of cells counted and analyzed based on the corrected dilution factor. In this experiment, the vehicle group (saline) was selected as the control group. The final results are expressed as the mean ± SD. The data were analyzed based on the value with the corrected dilution factor. For comparison of hematological indicators, unpaired two-tailed *t*-tests for multiple comparisons were used. The level of significance was set at *p* < 0.05. Statistical analyses were performed using Prism5 for Windows (GraphPad software, Inc., La Jolla, CA, USA).

### 4.14. In Vivo Tolerability Experiments

The CD-1 mice were purchased from Beijing Vital River Laboratory Animal Technology Co., Ltd.; each test group consisted of 6 animals (3 males and 3 females), and all animals were quarantined/adapted for at least 3 days prior to dosing. The CD-1 mice at 7–9 weeks of age, with an average weight (22–40 g males and 20–35 g females), were dosed via the tail vein with the prepared ADC (mil40-**15**: 10, 20, 40, 80, and 160 mg/kg) and the naked antibody (mil40: 10, 20, 40, 80, and 160 mg/kg) and a conventional valine-alanine (VA)-based cathepsin B-cleavable ADC (mil40-**16**: 10, 20, 40, 80, and 120 mg/kg) as controls. After administration, body weight changes of all test animals were monitored once a day, and the animal behaviors were observed twice a day next to a squirrel. The cage observation records include animal death or sudden death, the general health of the animal, and symptoms of drug toxicity. After the last observation, all surviving animals were euthanized by inhalation of 90–100% CO_2_.

### 4.15. Statistical Analysis

Data are expressed as means ± SD. For comparison of the above indicators, unpaired two-tailed *t*-test for multiple comparisons was used. The level of significance was set at *p* < 0.05. Statistical analyses were performed using Prism 8 for Windows (Graphpad software, Inc., La Jolla, CA, USA).

## 5. Conclusions

At present, the toxicity of ADC is still severely limiting its clinical benefits. Although the design of enzyme-cleavable linkers for MMAE-based ADCs has become more complex, it has not currently overcome the narrow therapeutic window. We reported an internalizable ADC using the ionized Cys-linker-MMAE as the payload. This ADC has high plasma stability and significant in vitro and in vivo efficacy; more importantly, it combines improved stability with reduced toxicity, which provides a potential strategy for further increasing the therapeutic index. For the first time, this study provides evidence that Cys-linker-MMAE can be used as a potent payload for ADCs. The design of this type of MMAE-based ADC is structurally simple, and this strategy is expected to provide a viable solution for significantly improving treatment safety.

## Figures and Tables

**Scheme 1:**
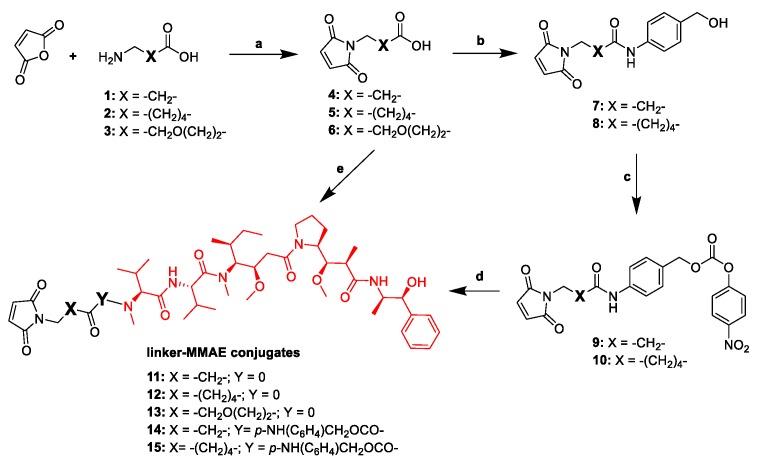
General synthetic route for the linker-MMAE conjugates. Reagents and conditions: (**a**) acetic acid, 120 °C, 6–8 h, 74%–86%; (**b**) *p*-aminobenzyl alcohol, 1-ethyl-(3-dimethylaminopropyl) carbodiimide hydrochloride (EDCI), *N*,*N*′-diisopropylethylamine (DIPEA), dichloromethane (DCM), room temperature (rt), overnight, 33%–55%; (**c**) bis(4-nitrophenyl) carbonate, DIPEA, *N*,*N*′-dimethylformamide (DMF), rt, 5 h, 63%~65%; (**d**) MMAE, 1-hydroxybenzotriazole (HOBt), DIPEA, DMF, rt, overnight, 50%~90%; (**e**) MMAE, diethyl cyanophosphonate, DIPEA, DCM, rt, overnight, 55%–63%.

**Table 1:** Structures of the synthetic linker-MMAE conjugates.

Linker-MMAE	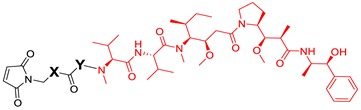	
	X	Y
**11**	-CH_2_-	-
**12**	-(CH_2_)_4_-	-
**13**	-CH_2_O(CH_2_)_2_-	-
**14**	-CH_2_-	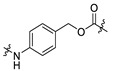
**15**	-(CH_2_)_4_-	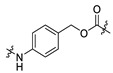
**16**	-(CH_2_)_4_-	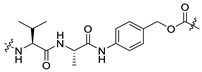

**Table 2:** Cytotoxicity of the Cys-linker-MMAE-based ADC in cancer cell lines in vitro.

Cell Lines	HER2 Status	mil40-15		mil40		MMAE		Cys-15	
		IC_50_ (nM)	Max. Inhibition	IC_50_ (nM)	Max. Inhibition	IC_50_(nM)	Max. Inhibition	IC_50_ (nM)	Max. Inhibition
BT-474	HER2^+++^	0.35	84.49%	0.78	65.45%	0.32	79.09%	6.88	82.62%
HCC1954	HER2^+++^	0.09	96.29%	1753.53	70.36%	0.07	97.54%	2.31	98.86%
NCI-N87	HER2^+++^	0.12	95.01%	0.19	87.90%	0.07	89.37%	8.67	94.86%
MCF-7	HER2^−^	110.54	88.06%	>3438.25	49.43%	0.29	93.14%	51.23	91.13%
MDA-MB-468	HER2^−^	97.62	87.42%	1388.43	59.13%	0.17	87.37%	11.11	85.75%

Note: “^+++^” indicates the antigen HER2 is positive; “^−^” indicates the antigen HER2 is negative.

## Supplementary Materials

The following are available online at https://www.mdpi.com/2072-6694/12/3/744/s1, Figure S1: Structures of cleavable linker systems for current MMAE-based ADCs, Figure S2: Drug release study of the Cys-linker-MMAE-based ADC at the cellular level, Figure S3: Flow cytometry for apoptosis and cell cycle arrest analysis, Figures S4–S28: The original mass spectrometry and NMR spectra details of the synthesized compounds **4**–**15**. Table S1: Permeability prediction of MMAE and Cys-linker-MMAE conjugates.
