# Supplementary material for: Antibody-Drug Conjugate Using Ionized Cys-Linker-MMAE as the Potent Payload Shows Optimal Therapeutic Safety
Source: Cancers (Basel). 2020 Mar 21;12(3):744. doi: 10.3390/cancers12030744 (PMC7140114; doi:10.3390/cancers12030744)
Supplement: Supplementary file 1 [file cancers-12-00744-s001.pdf]

cancers

MDPI

Article

Antibody-Drug Conjugate Using Ionized Cys-Linker-MMAE as the Potent Payload Shows Optimal Therapeutic Safety

Yanming Wang, Lianqi Liu, Shiyong Fan, Dian Xiao, Fei Xie, Wei Li, Wu Zhong and Xinbo Zhou

Supplementary Materials

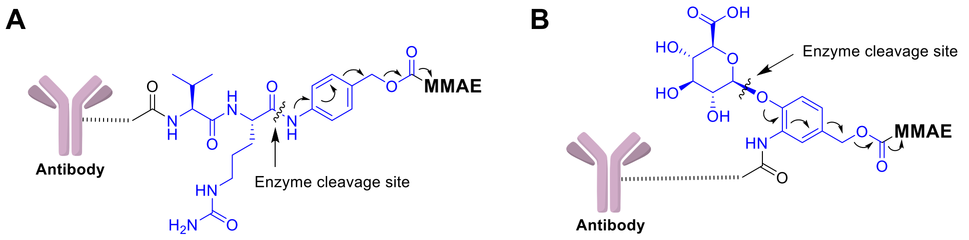

Figure S1. Structures of cleavable linker systems for current MMAE-based ADCs. (**A**) Structure of the cathepsin B-cleavable ADCs and its drug release pattern. (**B**) Structure of the β-glucuronidase-cleavable ADCs and its drug release pattern.

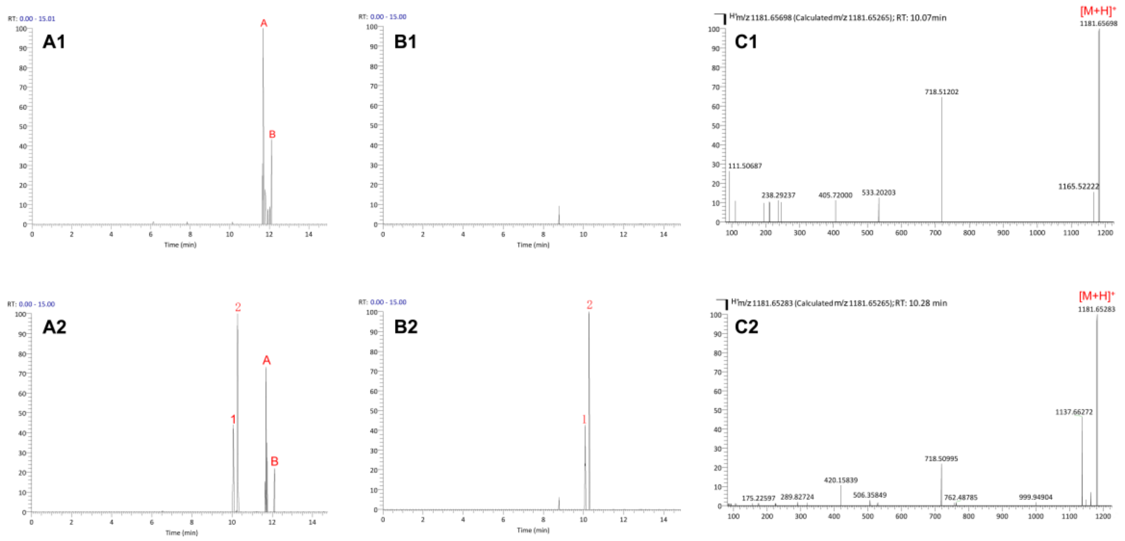

Figure S2. Drug release study of the Cys-linker-MMAE-based ADC at the cellular level. (**A1**) The XIC of full MS/ddMS2 scan of the vehicle in BT-474 cells; (**A2**) The XIC of full MS/ddMS2 scan of the metabolites in BT-474 cells; (**B1**) The XIC of full MS/ddMS2 scan of the vehicle in NCI-N87 cells; (**B2**) The XIC of full MS/ddMS2 scan of the metabolites in NCI-N87 cells; (**C1**) The MS2 fragmentation of M1 (R.T. = 10.07 min); (**C2**) The MS2 fragmentation of M2 (R.T. = 10.28 min). The compound A (m/z 591.32976, R.T=11.69 min) and B (m/z 591.32976, R.T = 12.11 min) could also be detected in the control group of BT-474 cells (Figure A1 and A2), meaning that they are not related to the ADC being administered. Each dosing group included two replicates. R.T.: retention time.

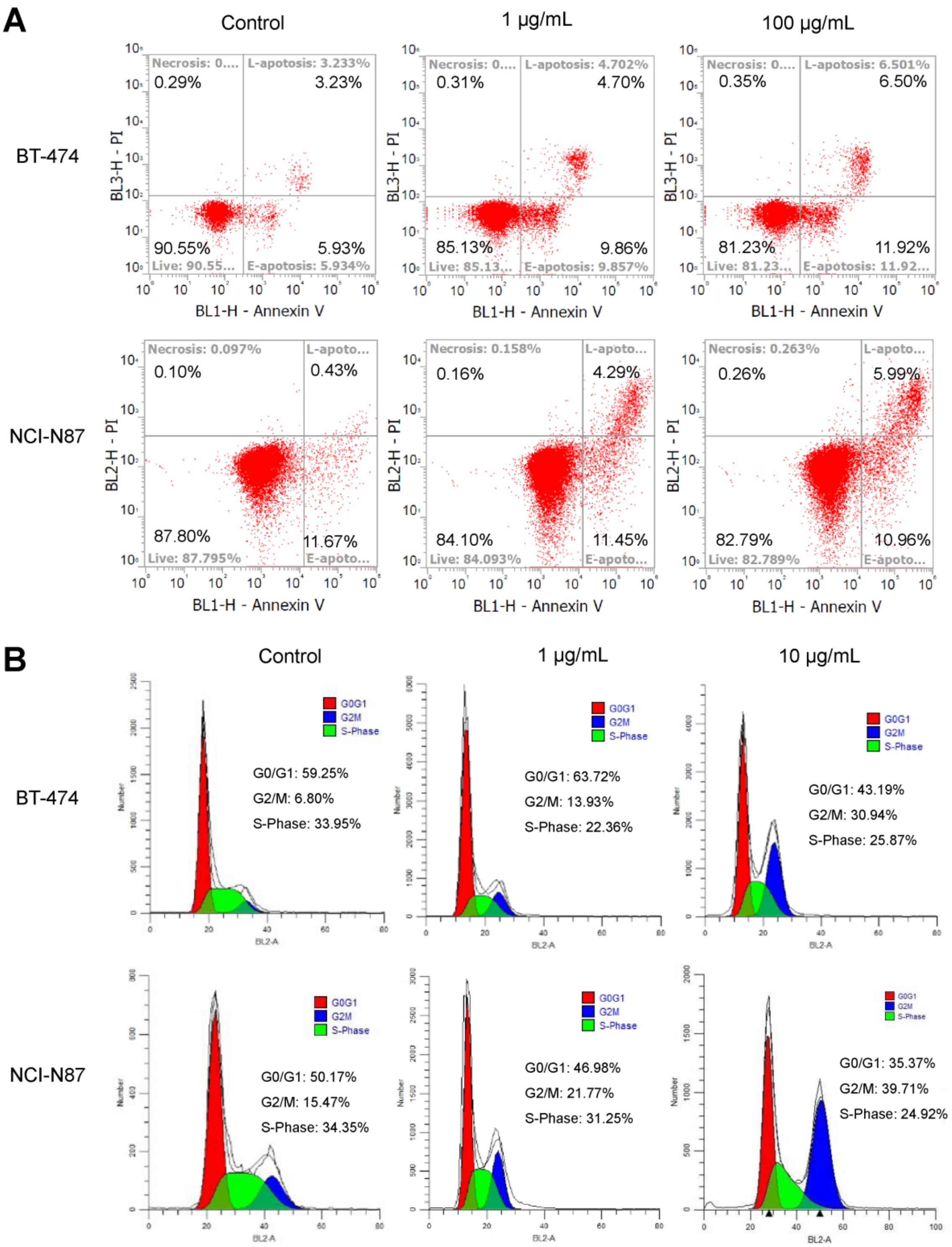

Figure S3. Flow cytometry for apoptosis and cell cycle arrest analysis. (**A**) The induction of apoptosis in BT-474 and NCI-N87 cells was detected by flow cytometry; the cells were treated with mil40-**15** for 24 h. (**B**) Cell cycle arrest analysis in the BT-474 and NCI-N87 cells was detected by flow cytometry; the cells were treated with mil40-**15** for 24 h.

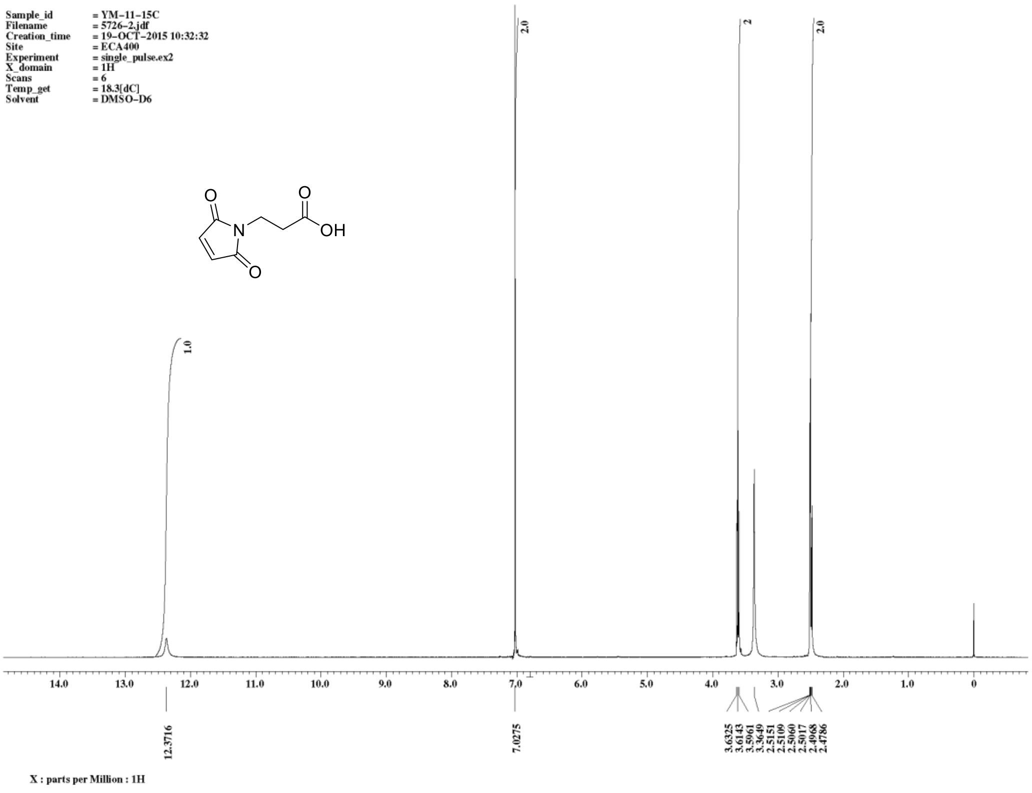

Figure S4. The ^{1}H-NMR spectrum of compound **4.**

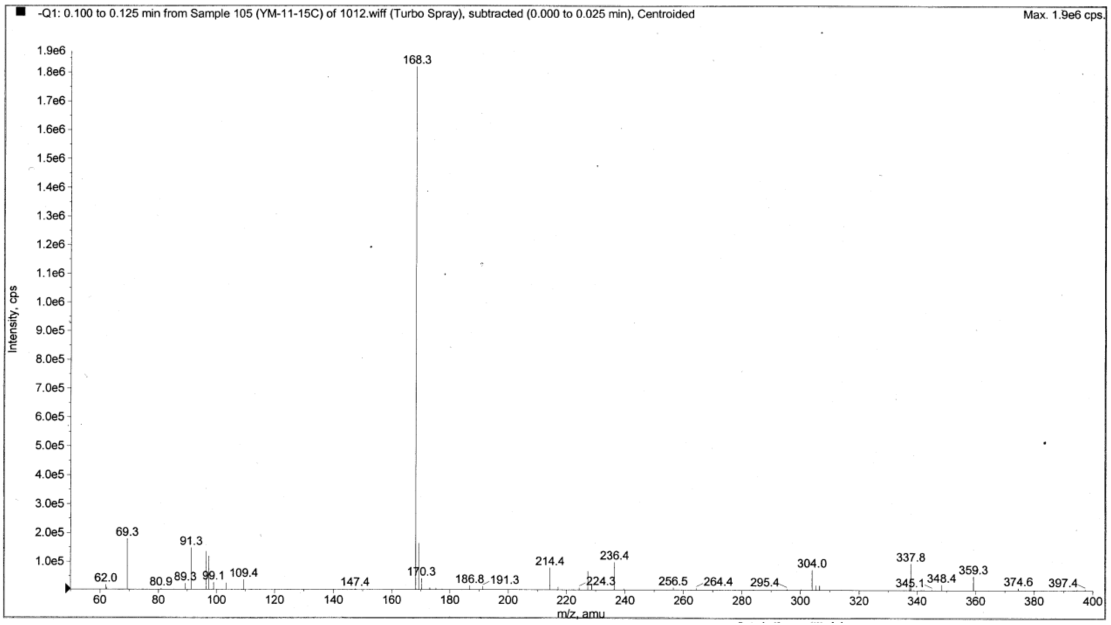

Figure S5. The ESI-MS spectrum of compound **4**.

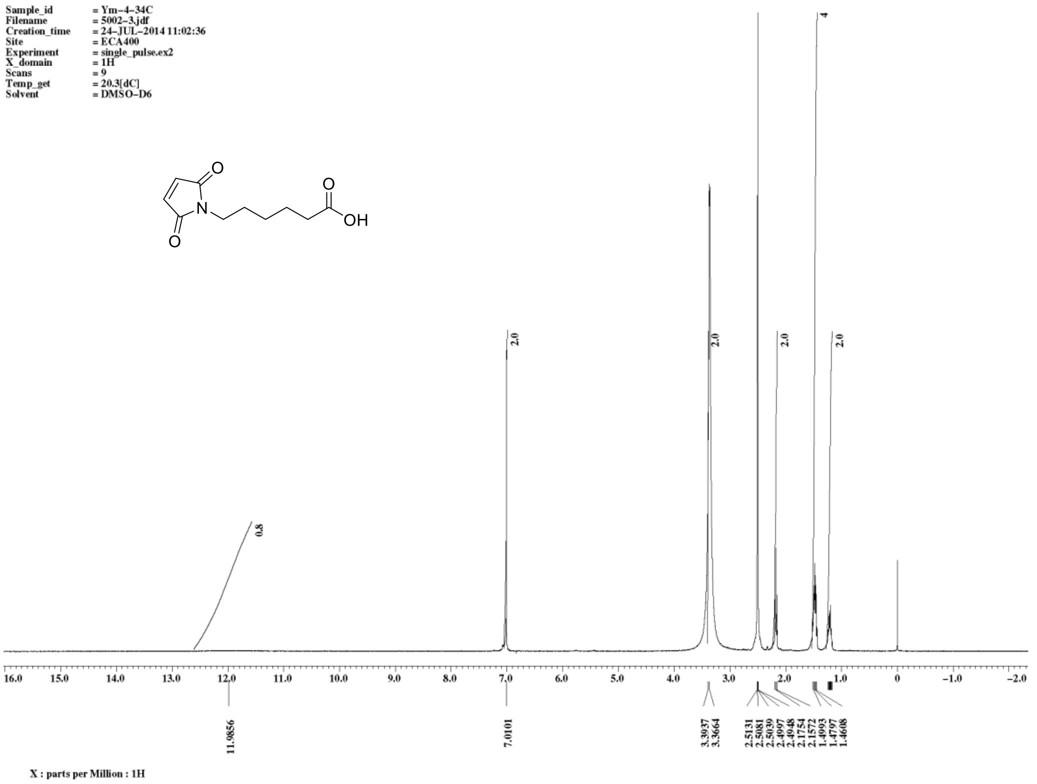

Figure S6. The ^{1}H-NMR spectrum of compound **5**.

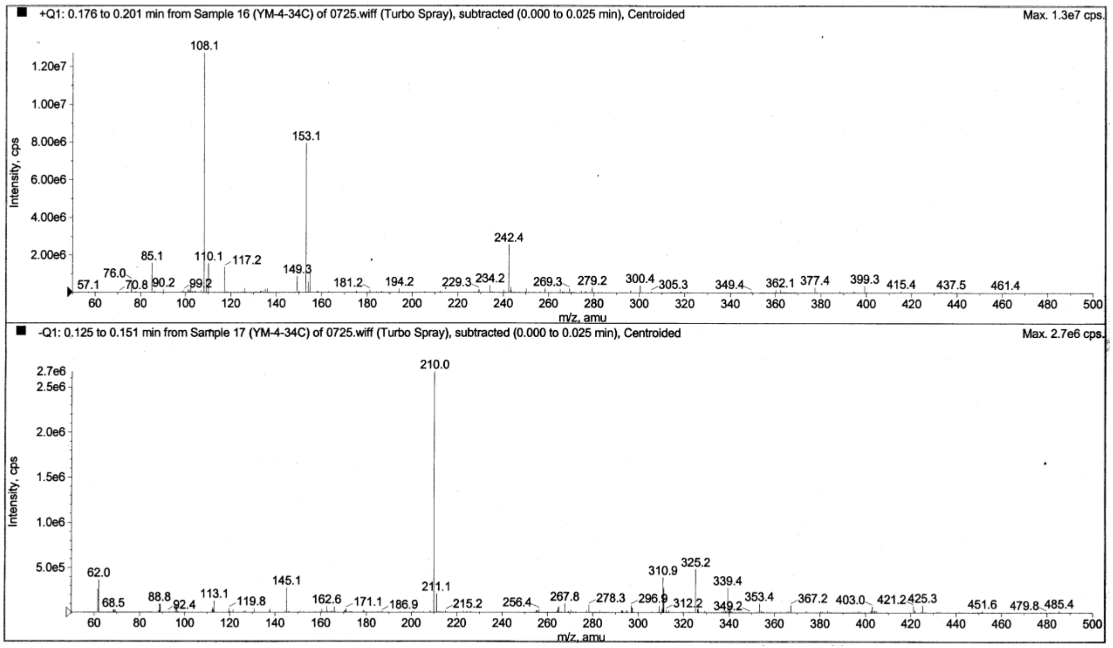

Figure S7. The ESI-MS spectrum of compound **5**.

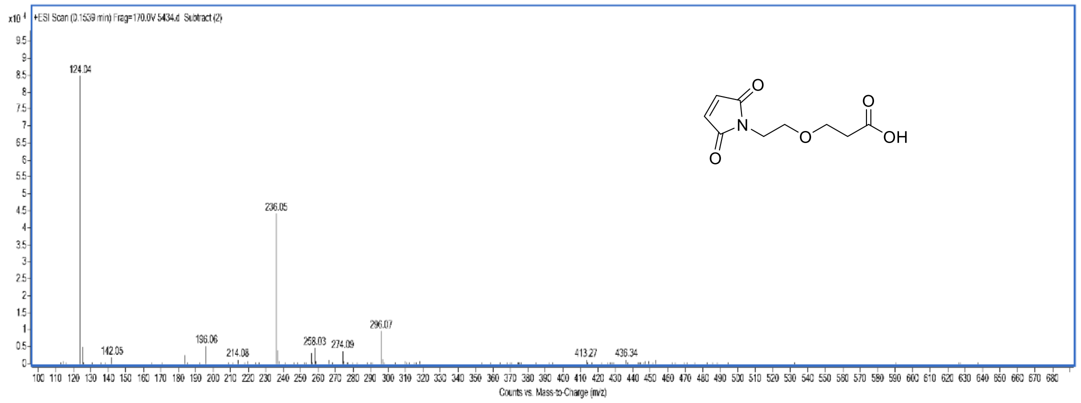

Figure S8. The ESI-MS spectrum of compound **6**.

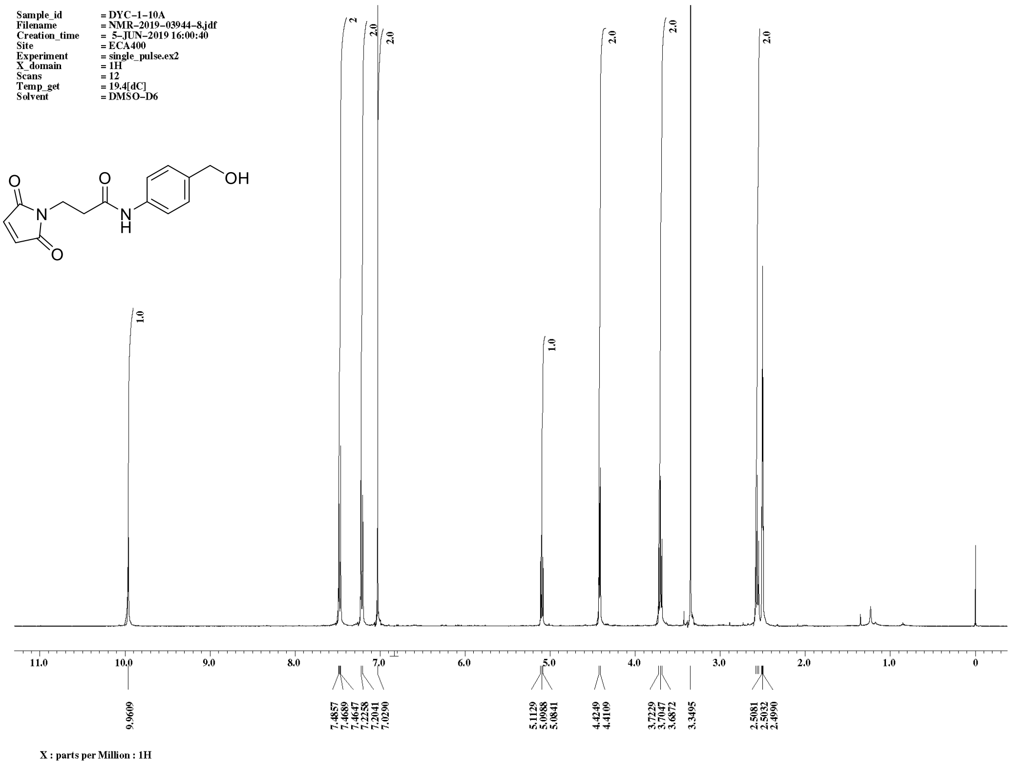

Figure S9. The ^{1}H-NMR spectrum of compound **7**.

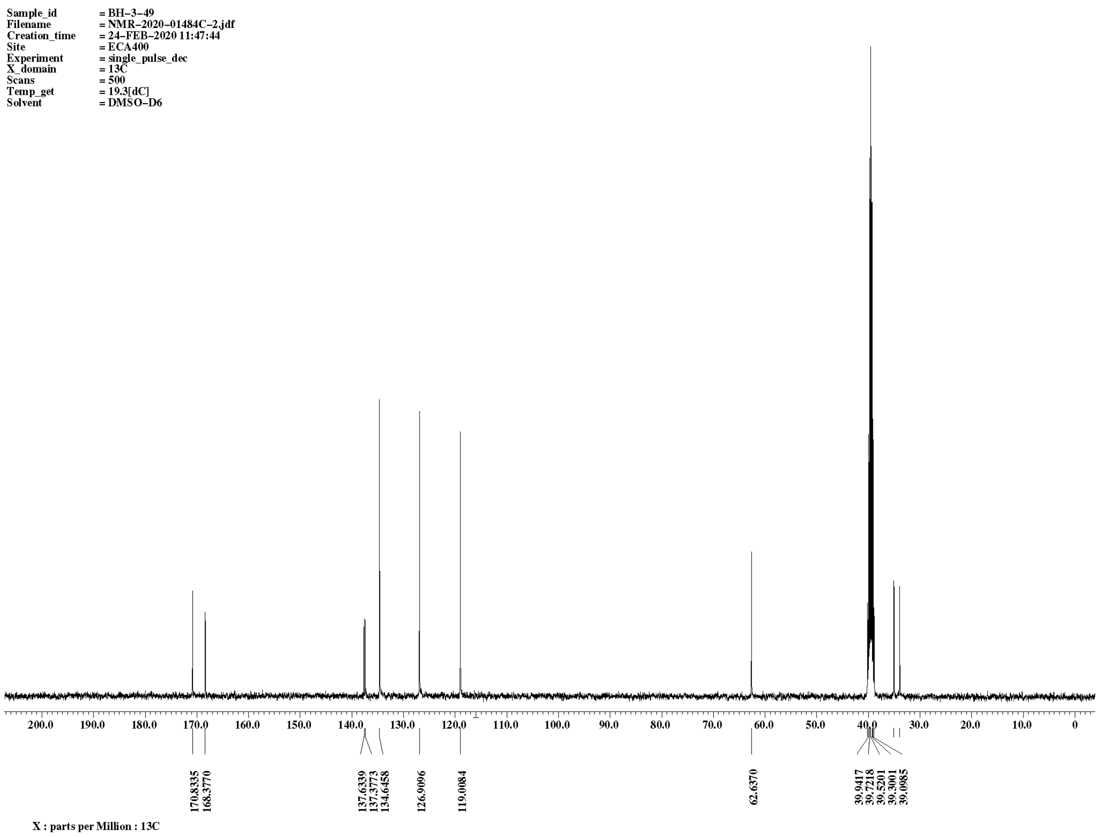

Figure S10. The ^{13}C-NMR spectrum of compound **7**.

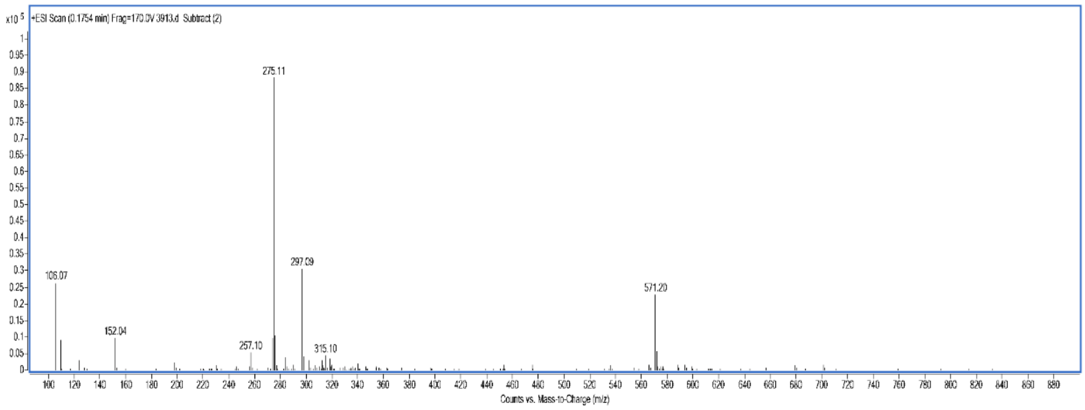

Figure S11. The ESI-MS spectrum of compound **7**.

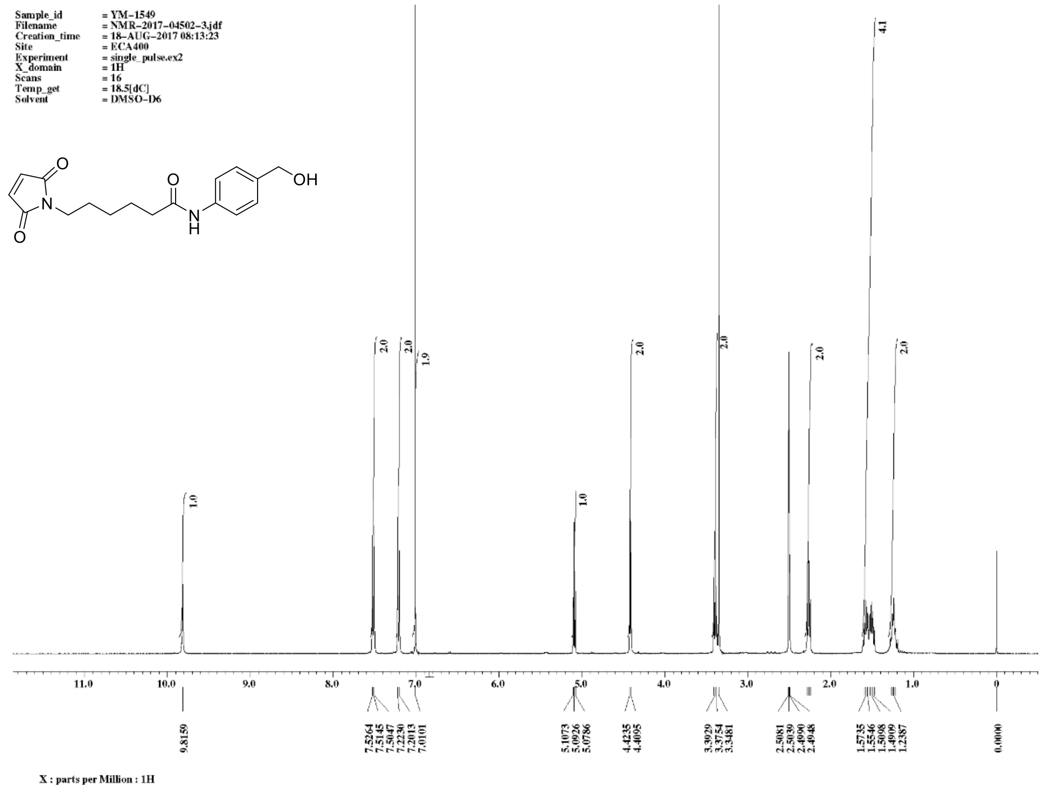

Figure S12. The ^{1}H-NMR spectrum of compound **8**.

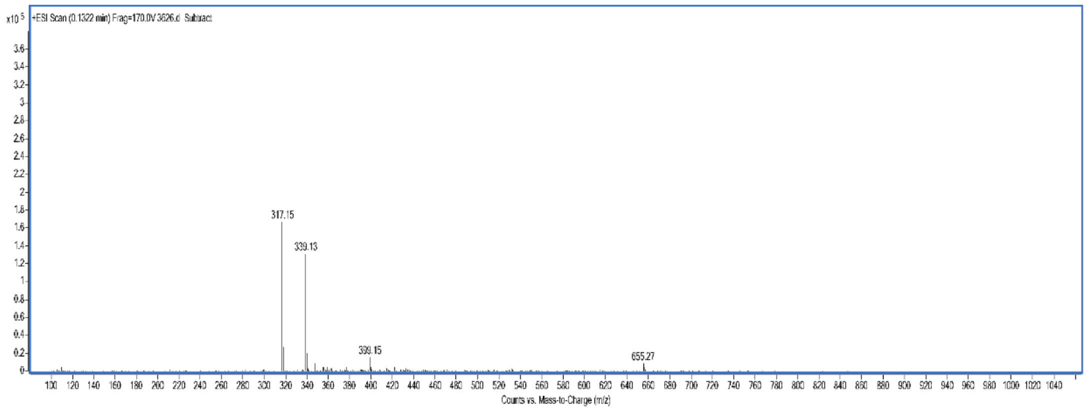

Figure S13. The ESI-MS spectrum of compound **8**.

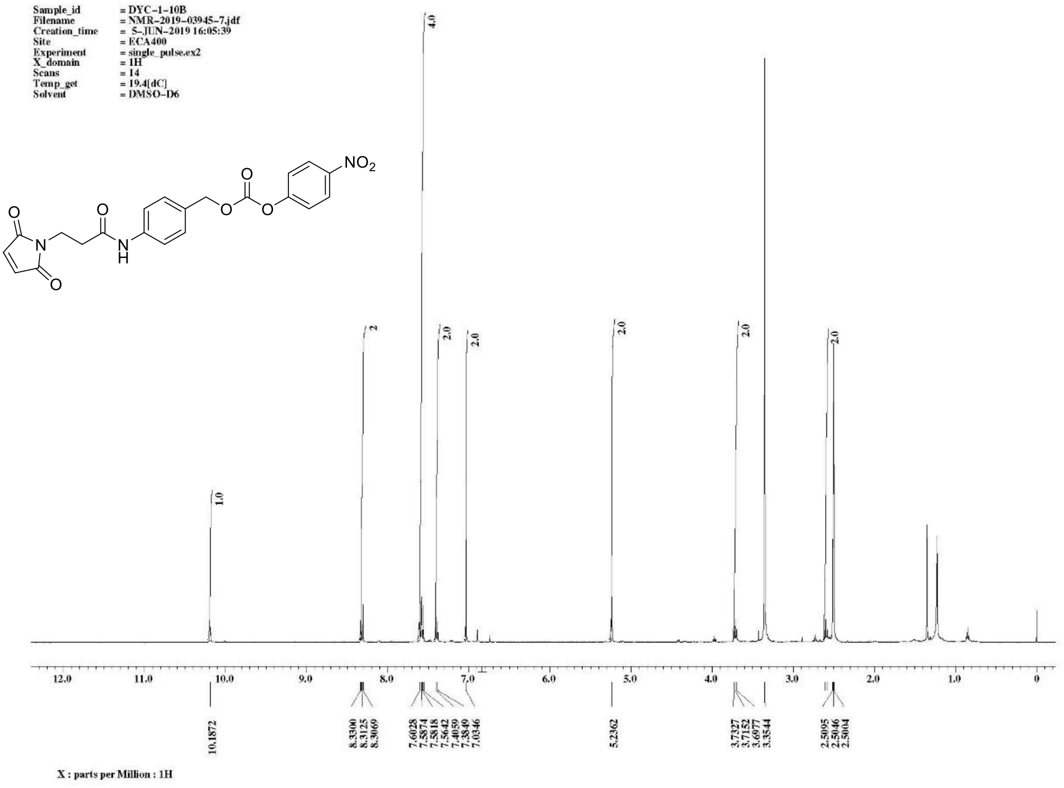

Figure S14. The ^{1}H-NMR spectrum of compound **9**.

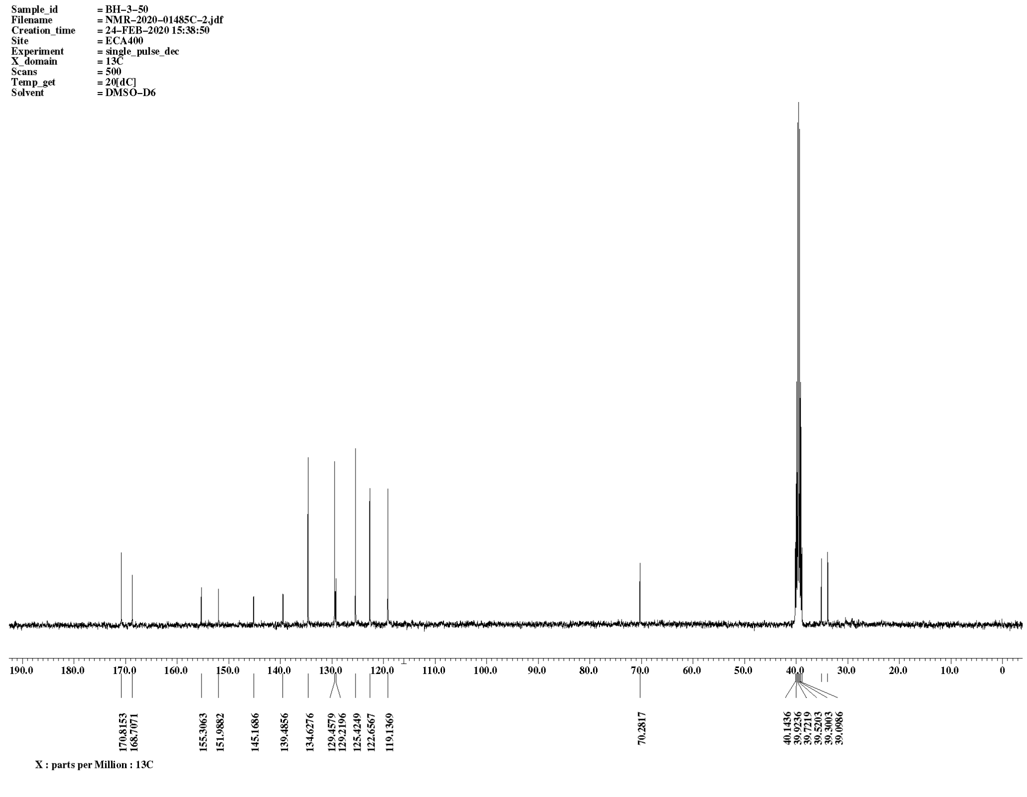

Figure S15. The ^{13}C-NMR spectrum of compound **9**.

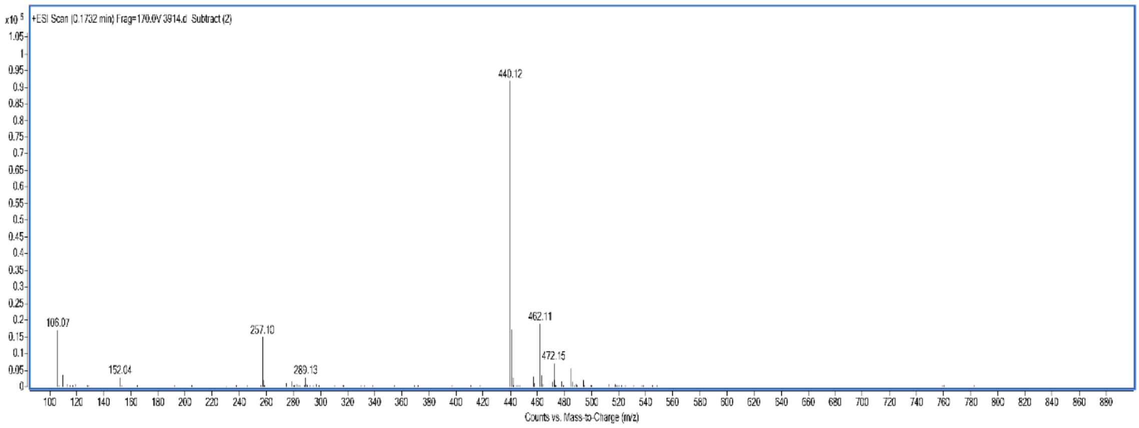

Figure S16. The ESI-MS spectrum of compound **9**.

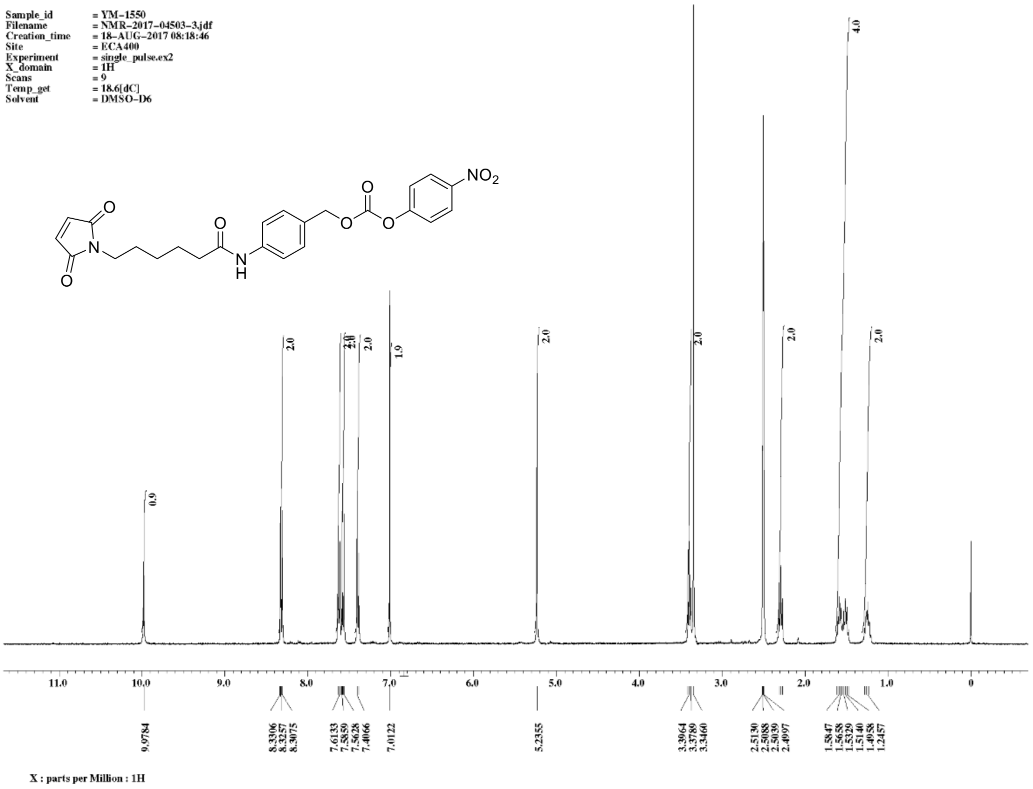

Figure S17. The ^{1}H-NMR spectrum of compound **10**.

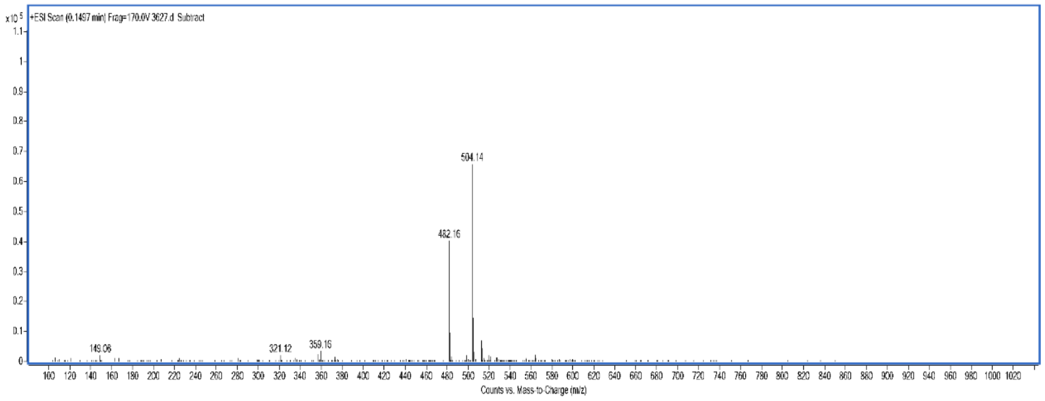

Figure S18. The ESI-MS spectrum of compound **10**.

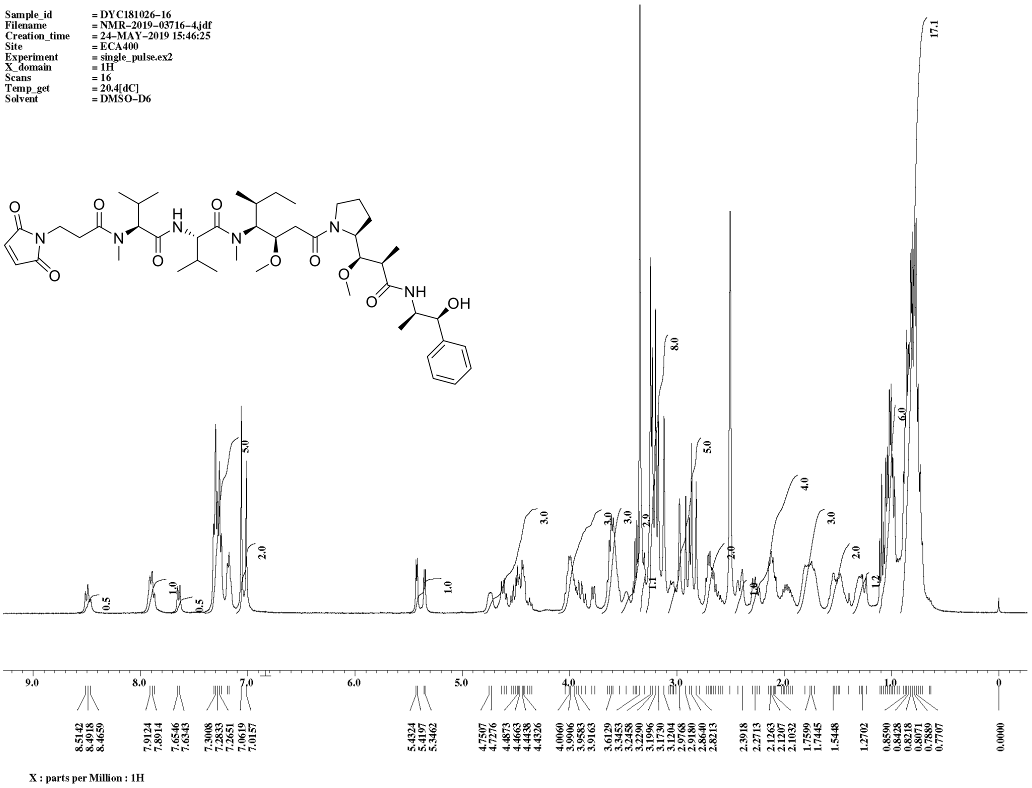

Figure S19. The ^{1}H-NMR spectrum of compound **11**.

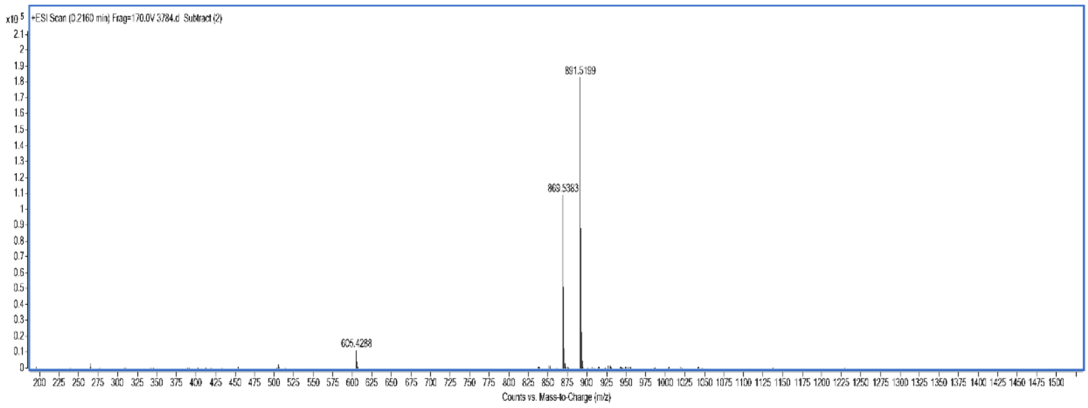

Figure S20. The HRMS spectrum of compound **11**.

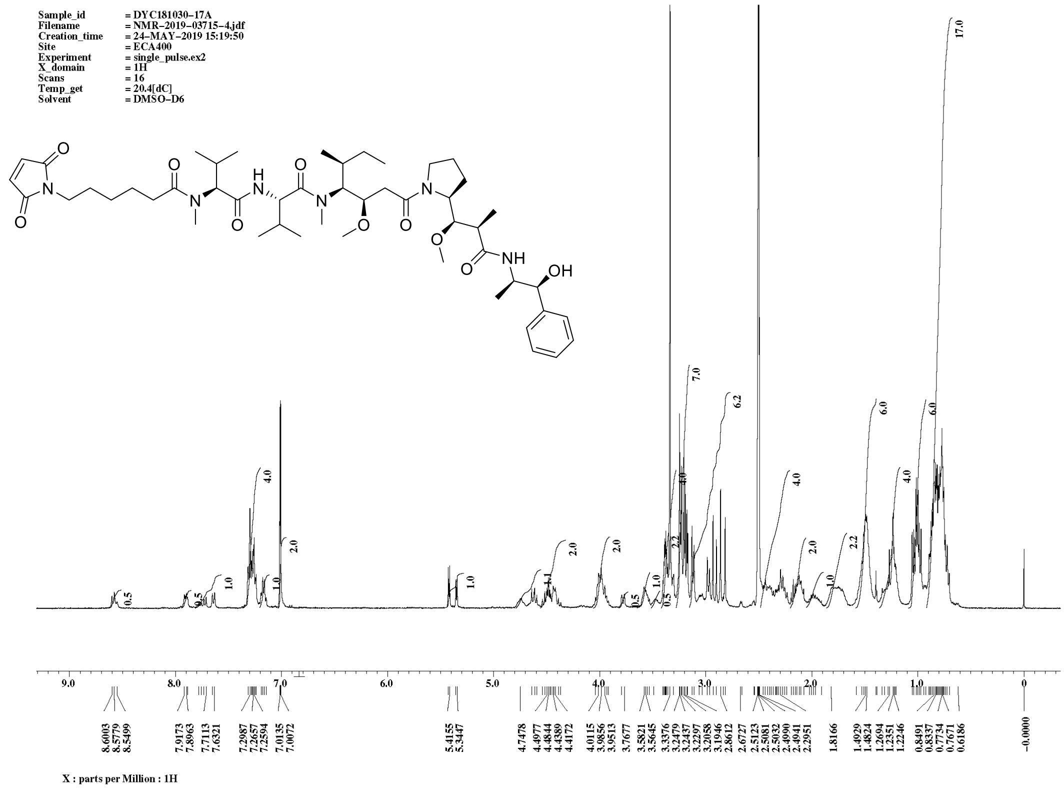

Figure S21. The ^{1}H-NMR spectrum of compound **12**.

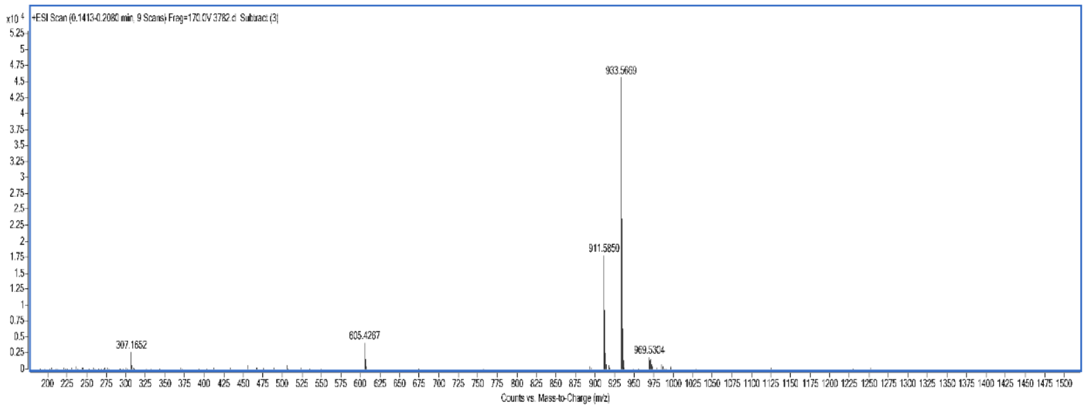

Figure S22. The HRMS spectrum of compound **12**.

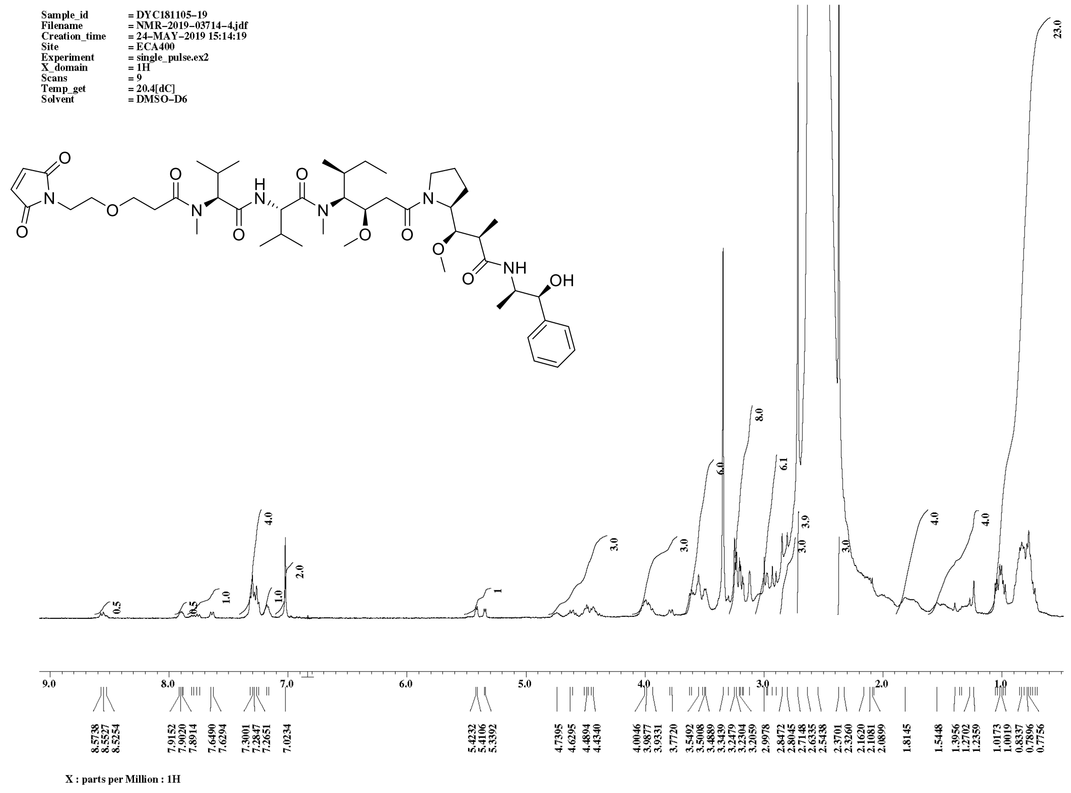

Figure S23. The ^{1}H-NMR spectrum of compound **13**.

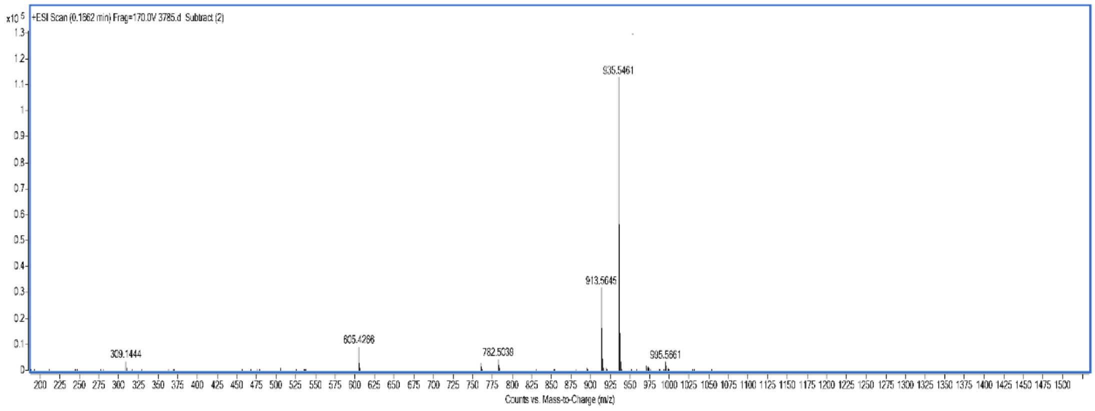

Figure S24. The HRMS spectrum of compound **13**.

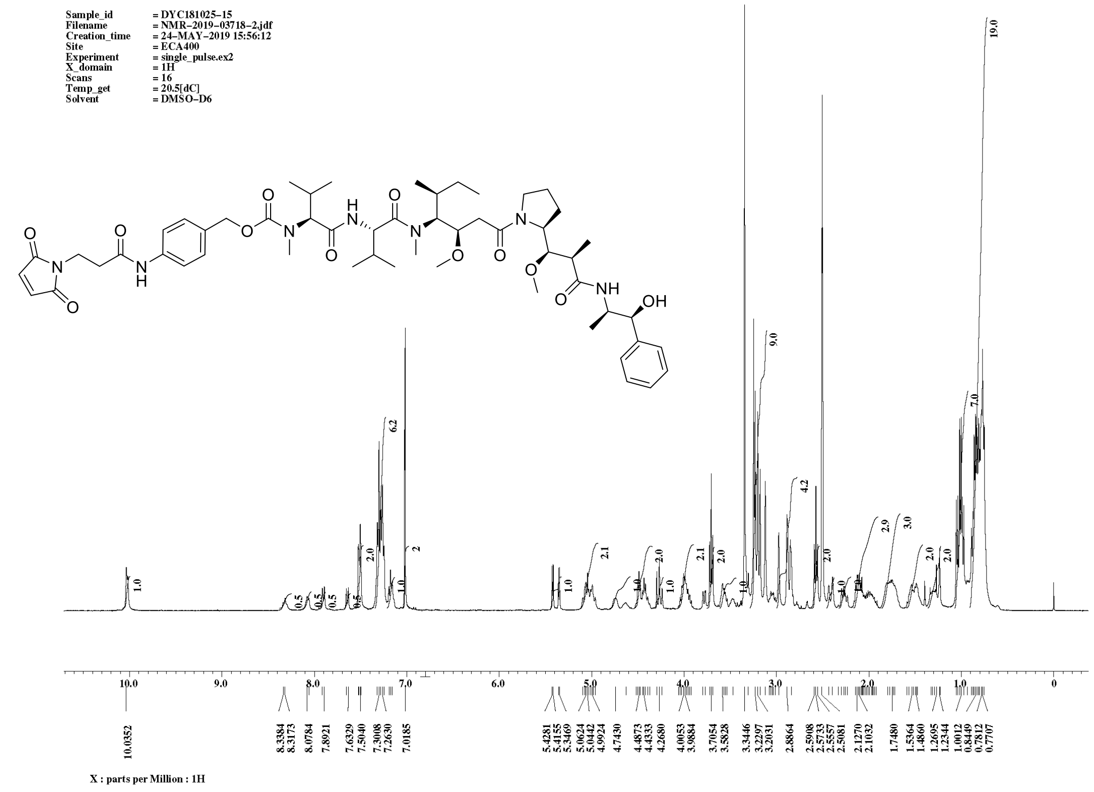

Figure S25. The ^{1}H-NMR spectrum of compound **14**.

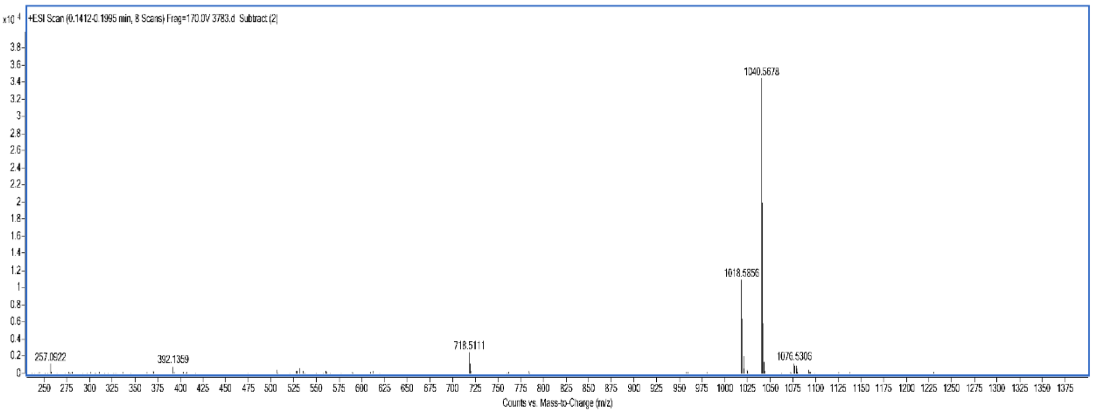

Figure S26. The HRMS spectrum of compound **14**.

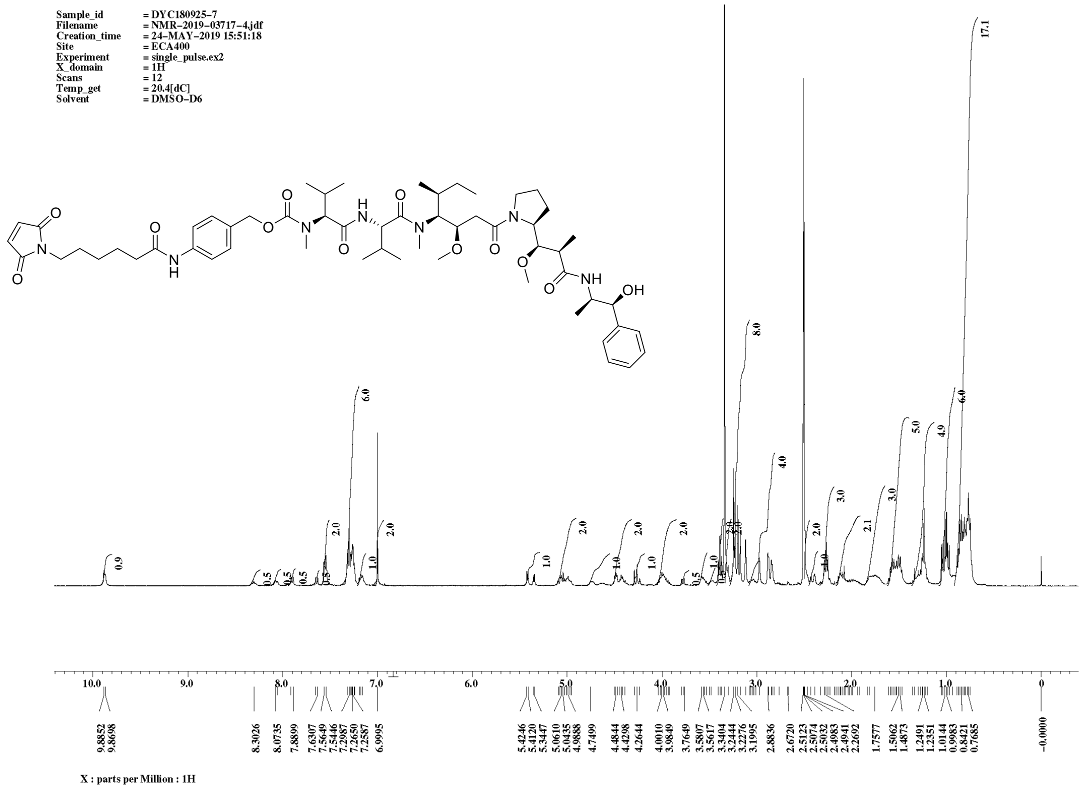

Figure S27. The ^{1}H-NMR spectrum of compound **15**.

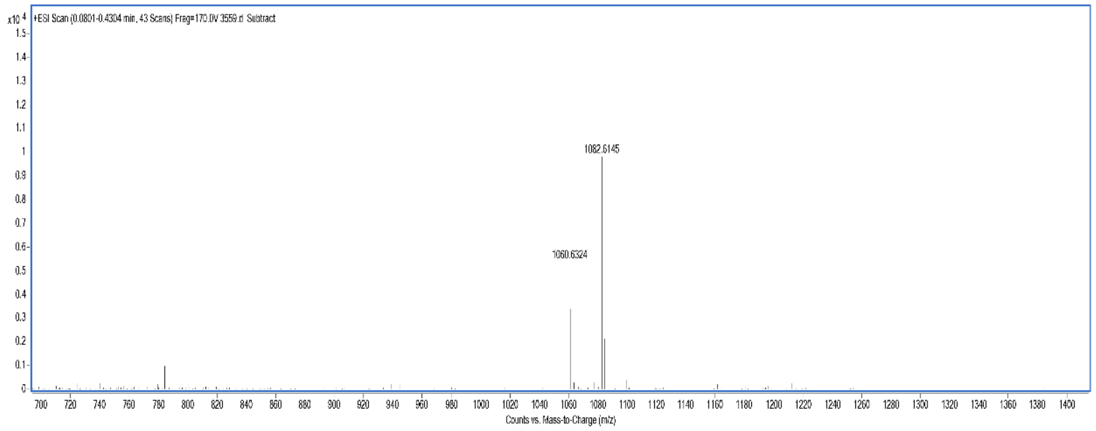

Figure S28. The HRMS spectrum of compound **15**.

Table S1. Permeability prediction of MMAE and Cys-linker-MMAE conjugates.

**Compound**	**MlogP**	**S + Peff (cm/s × 10^4)**	**S + MDCK (cm/s × 10^7)**	**Perm Cornea (cm/s × 10^7)**
Cys-**11**	−2.931	0.176	5.747	10.399
Cys-**12**	−2.464	0.190	4.902	11.843
Cys-**13**	−3.299	0.168	4.906	10.573
Cys-**14**	−2.533	0.162	5.019	12.262
Cys-**15**	−2.093	0.181	4.795	13.842
MMAE	1.191	0.353	21.818	63.090

MlogP: moriguchi model of octanol-water partition coefficient, larger logP values indicate higher lipophilicity; S + Peff: human effective jejunal permeability, larger S + Peff values indicate greater permeability and the predicted value for membrane permeable molecules is usually >0.25; S + MDCK: apparent MDCK COS permeability, larger S + MDCK values also indicate greater permeability and the predicted value for membrane permeable molecules is usually >20; Perm_Cornea: permeability through rabbit cornea, larger Perm_Cornea values indicate greater membrane permeability.

© 2020 by the authors. Licensee MDPI, Basel, Switzerland. This article is an open access article distributed under the terms and conditions of the Creative Commons Attribution (CC BY) license (http://creativecommons.org/licenses/by/4.0/).
